# Human intestinal parasites in Mahajanga, Madagascar: The kingdom of the protozoa

**DOI:** 10.1371/journal.pone.0204576

**Published:** 2018-10-10

**Authors:** Valentin Greigert, Ahmed Abou-Bacar, Julie Brunet, Céline Nourrisson, Alexander W. Pfaff, Leila Benarbia, Bruno Pereira, Milijaona Randrianarivelojosia, Jean-Louis Razafindrakoto, Rivo Solotiana Rakotomalala, Eugène Morel, Ermanno Candolfi, Philippe Poirier

**Affiliations:** 1 Service de médecine interne, Hôpitaux Civils de Colmar, Colmar, France; 2 Institut de Parasitologie et Pathologie Tropicale, Université de Strasbourg, Strasbourg, France; 3 Laboratoire de Parasitologie-Mycologie, Plateau Technique de Microbiologie, CHU de Strasbourg, Strasbourg, France; 4 Laboratoire de Parasitologie-Mycologie, CHU Clermont-Ferrand, Clermont-Ferrand, France; 5 Université Clermont Auvergne, CNRS, Laboratoire Microorganismes: Génome et environnement (LMGE), Clermont–Ferrand, France; 6 Unité de Biostatistiques, Direction de la Recherche Clinique (DRCI), CHU de Clermont-Ferrand, France; 7 Laboratoire d’Analyse Médicale, CHU PZaGa, Mahajanga, Madagascar; 8 Service d’Hépato-Gastro-Entérologie, CHU Mahavoky Atsimo, Mahajanga, Madagascar; NIH, UNITED STATES

## Abstract

**Introduction:**

Intestinal parasitic infections are a major public health problem in inter-tropical areas. The aim of our study was to describe the situation in Mahajanga, Madagascar with a particular focus on two protozoa, *Dientamoeba fragilis* and *Blastocystis* sp.

**Methods:**

This was a prospective study from February to June 2015. Stool samples from symptomatic hospitalized patients and asymptomatic volunteers were submitted to microscopy and molecular assays in order to detect parasites.

**Results:**

A wide panel of intestinal parasites were identified among the 265 included subjects, protozoa being the most prevalent with 72.8% whereas the prevalence of helminths and microsporidia was of 7.9% and 4.5%, respectively. *Blastocystis* sp. was the most prevalent protozoa (64.5% of the entire cohort) followed by various amoebas (35.5%) and flagellates (27,5%). We only detected subtypes 1, 2 and 3 of *Blastocystis* sp. Among the patients positive for *D*. *fragilis* (9.4%), 23 carried genotype 1 and 1 genotype 2. For the first time, we detected in 4 human stools the DNA of a recently described protozoon, *Simplicimonas similis*. Interestingly, subjects living in urban areas harbored significantly more different parasitic species than subjects living in rural areas with a correlation between sanitary level of neighborhood and protozoan infection. However, there was no difference in prevalence of digestive symptoms between parasite-free and parasite-infected subjects, except for *Giardia intestinalis* which had more symptomatic carriers.

**Discussion:**

Our study reveals a high overall parasite prevalence, similar to what had been found in 2003 in the same city and to other prevalence studies conducted in Africa. The poor access of the population to sanitary infrastructures may explain this result. Data from our study provide valuable key for sanitation programs and prevention of fecal-related infectious diseases.

## Introduction

Health issues in the intertropical regions are dominated by three major diseases: HIV infection, malaria and tuberculosis. Intestinal parasitic infections are nevertheless a major public health problem in areas where favorable weather, insufficient hygiene conditions and poverty favor their persistence. In 2010, the publication of the Global Burden of Disease Study 2010 (GBD 2010) showed once again the dramatic burden of these infections, with more than 10 million disability-adjusted life year (DALYs) worldwide but mostly in developing countries [1,2]. Only few recent publications describe the overall impact of intestinal parasitic infections in populations living in intertropical areas, despite the fact that they are incriminated in various pathologic conditions such as asthma [3], troubles in child development [4], alteration of cognitive abilities [5] or malnutrition [6,7]. Regarding these data, very few information are available for Madagascar. However, in 2015, less than 45% Malagasy had access to clean drinking water and diarrhea was still the fourth cause of mortality (http://www.who.int/countries/mdg/en/). Moreover, soil transmitted helminths are well recognized to have an impact on health, in particular on stunting [4].

To our knowledge, only one study in 2003 made a sanitary state of parasitic intestinal infections in the Malagasy town of Mahajanga [8]. The aim of our study was to describe the situation of the same population twelve years later. We also put a focus on the emerging parasites *Blastocystis* sp. and *Dientamoeba fragilis*, for whom very few data in Africa are available.

## Material and methods

### Ethics statement

This study was approved by the Malagasy Ministry of Public Health (reference number 33-MSANP/CE). Written informed consents were obtained from all participants. This study was conducted in accordance with the Code of Ethics of the World Medical Association (Declaration of Helsinki).

### Questionnaire survey

A questionnaire recording the socioeconomic, demographic, alimentary habits and health conditions, including treatments, medical history, symptoms and Body Mass Index (BMI), was completed for each included subject.

### Study population and sample collection

This prospective study was conducted from February to June 2015 in Mahajanga, North-West Madagascar (15° 43’ 0" South, 46° 19’ 0" East), and the rural neighborhood. The subjects were recruited among patients hospitalized in the Department of Gastroenterology of the university hospital PZaGa of Mahajanga for exploration and treatment of digestive disorders, and volunteers from the city. Subjects under 18 years of age were excluded according to the authorization delivered by the Malagasy Ministry of Public Health. One stool sample was collected for each included subject. Treatment was proposed to positive patients for a parasite considered pathogenic.

### Parasitological examination

All fecal samples were submitted to microscopic examination in the 30 minutes following stool emission. Parasitological examination was performed by direct microscopic examination of fecal smear in saline solution, merthiolate-iodine-formalin (MIF) staining and Faust concentration method using zinc sulfate solution at a density of 1.118 g/ml [9,10]. *In vitro* protozoa cultures using an aliquot of each stool sample were performed using Dobell and Laidlaw biphasic medium (coagulated horse serum slant overlaid with Ringer’s solution supplemented with horse serum, penicillin-streptomycin and 2 g of rice starch) incubated at 37°C [11]. Screening of protozoa cultures was performed using standard light microscopy after 24 and 48h of culture. Stool samples were considered positive if helminth eggs, larvae, cysts and/or trophozoites of protozoans were detected by at least one of the four conventional methods and/or a positive molecular diagnosis (see below).

### Molecular diagnosis

We performed molecular diagnosis for a few parasite species in order to compare our results with recent studies using similar techniques, and to improve sensitivity and specificity of diagnosis, in particular regarding *Blastocystis* sp. or *D*. *fragilis* (Table 1). Thus, an aliquot of each stool sample was stored at -20°C until molecular diagnosis. DNA extractions were performed using the DNA Stool minikit (Qiagen) according to manufacturer recommendations.

**Table 1:** Molecular diagnosis methods and primers used in the present study. Endpoint values are indicated for each qPCR.

Parasite	Method	Primers	Endpoint value (Ct)	Ref.
*Blastocystis* sp.	qPCR (Sybr green)	BL18SPPF1: 5’-AGTAGTCATACGCTCGTCTCAAA-3’ BL18SPPR2: 5’-TCTTCGTTACCCGTTACTGC-3’	45	[12]
*Simplicimonas similis*	qPCR + sequencing	SSF: 5’-TGCGATTGTTTCACCGGATTACGA-3’ SSR: 5’- AACCATAAGGTTGACATGGAACTGGTC-3’	40	
*Cryptosporidium* sp.	qPCR (*Taq*Man)	F: 5’-CATGGATAACCGTGGTAAT-3’ R: 5’-TACCCTACCGTCTAAAGCTG-3’ Probe: 5’-FAM-CTAGAGCTAATACATGCGAAAAAA- BHQ1-3’	45	[16]
*Enterocytozoon bieneusi*	qPCR (*Taq*Man)	FEB1: 5’-CGCTGTAGTTCCTGCAGTAAACTATGCC-3’ REB1: 5’-CTTGCGAGCGTACTATCCCCAGAG-3’ Probe: 5’-FAM-CGTGGGCGGGAGAAATCTTAGTGTTCGGG-BHQ1-3’	45	[17]
*Encephalitozoon intestinalis*	qPCR (*Taq*Man)	FEI1: 5’-GCAAGGGAGGAATGGAACAGAACAG-3’ REI1: 5’-CACGTTCAGAAGCCCATTACACAGC-3’ Probe: 5’-FAM-CGGGCGGCACGCGCACTACGATA-BHQ1-3’	45	[18]
*Dientamoeba fragilis*	qPCR (*Taq*Man)	DF3: 5’-GTTGAATACGTCCCTGCCCTTT-3’ DF4: 5’-TGATCCAATGATTTCACCGAGTCA-3’ Probe: 5’-FAM-CACACCGCCCGTCGCTCCTACCG-BHQ1-3’	40	[13]
	nested PCR + sequencing	1^st^ pair: TRD5: 5’-GATACTTGGTTGATCCTGCCAAGG-3’ TRD3: 5’-GATCCAACGGCAGGTTCACCTACC-3’ 2^nd^ pair: DF1: 5’-CTCATAATCTACTTGGAACCAATT-3’ DF4: 5’-CCCCGATTATTCTCTTTGATATT-3’		[13] [14] [15]
*E*. *histolytica*/*E*. *dispar*	PCR	ED1: 5’-TACAAAGTGGCCAATTTATGTAAGTA-3’ EH1: 5’-GTACAAAATGGCCAATTCATTCAATG-3’ EHD2: 5’-ACTACCAACTGATTGATAGATCAG-3’		[19]
Hookworms	PCR + sequencing	RTHW1F: 5’-GATGAGCATTGCWTGAATGCCG-3’ RTHWR: 5’-GCAAGTRCCGTTCGACAAACAG-3’		[20]

Previously published end-point or real-time PCR (Table 1) were used for the diagnosis of *Blastocystis* sp. [12], *D*. *fragilis* [13–15], *Cryptosporidium* sp. [16], *Enterocytozoon bieneusi* [17] and *Encephalitozoon intestinalis* [18], *Entamoeba histolytica/dispar* [19] and hookworms [20]. Subtyping of *Blastocystis* sp. was performed by sequencing real-time PCR products as previously described [12]. Genotyping of *D*. *fragilis* isolates was carried out by nested PCR and sequencing a fragment of the gene encoding for the small ribosomal subunit [14], followed a second PCR using *D*. *fragilis*-specific primers pair DF1/DF4 [15]. The sequences obtained were submitted to the NCBI database using the nucleotide basic local alignment search tool (http://www.ncbi.nlm.nih.gov/BLAST/) and genotypes were identified by determining the exact match or closest similarity against genotype 1 or genotype 2 (strain Bi/Pa) [21]. *Blastocystis* sp. subtypes (STs) were identified by determining the exact match or closest similarity against all known STs, according to the updated classification of Alfellani *et al*. [22]. Specific primers were designed to amplify a 260 bp fragment length of the small ribosomal subunit of *Simplicimonas similis*. All patients included in the study were screened for *S*. *similis*. Real-time PCR was carried out on a LightCycler 2.0 (Roche Diagnostics) in a final volume of 20 μl containing 2μl of LC-FastStart DNA Master SYBR green kit (Roche Diagnostics, France), 5 mM MgCl_2_, 0.5 μM each primer (SSF 5’-TGCGATTGTTTCACCGGATTACGA-3’; SSR 5’-AACCATAAGGTTGACATGGAACTGGTC-3’), and 2 μl of DNA template. After denaturing at 95°C for 10 minutes, 45 cycles were run with 5 s of denaturation at 95°C, 10 s of annealing at 68°C, and 15 s of extension at 72°C. PCR products of all positive samples were subsequently sequenced.

### Statistical analyses and figures

Statistical analyses were performed using RStudio and QtiPlot softwares. The tests were two-sided, with a type I error set at α = 0.05. Quantitative data was presented as means ± standard deviation. The categorical data was presented as frequency and associated proportions. The differences across groups were compared using Student’s t-test and the chi-squared test or Fisher’s exact test for categorical parameters. Figures were elaborated using LibreOffice Calc, QtiPlot and TheGIMP softwares. Cartography base were obtained using OpenStreetMap (OpenStreetMap contributors), kindly provided by Geofabrik under CC-BY 4.0.

## Results

### Study population

A total of 265 subjects were recruited with a mean age of 32.8±14.5 years and sex ratio of 0.8 (118 M/147 F). Sixty-seven patients complained of digestive symptoms, with a mean age of 35.2±15.2 years and a sex ratio of 1.31. For asymptomatic subjects (n = 198), mean age was 31.9±14.2 years and the sex ratio of 0.68. The BMI was significantly higher for asymptomatic subjects (22.04 kg/m^2^) than for symptomatic ones (20.74 kg/m^2^) (*p*<0.01). In the symptomatic group, symptoms were mostly abdominal pain (64.2%; n = 43/67), diarrhea (46.3%; n = 31/67) and bloated feeling (37.3%; n = 25/67).

### Geographical features

**Fig 1:**
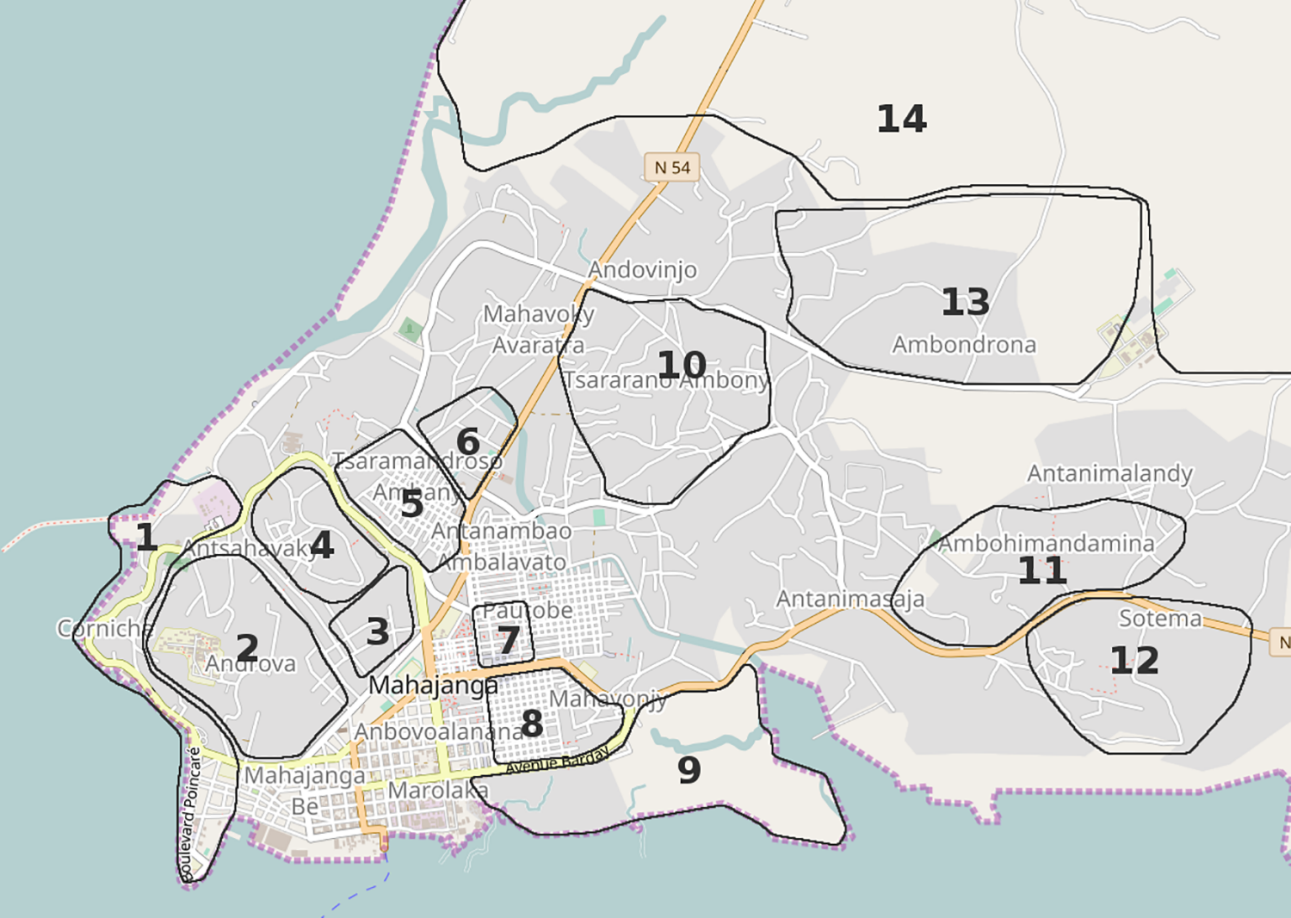
Mahajanga districts. Numbers are corresponding to: 1. Corniche; 2. Androva; 3. Mangarivotra; 4. Antsahavaky; 5. Tsaramandroso ambany; 6. Tsaramandroso cité; 7. Morafeno; 8. Abattoir; 9. Aranta; 10. Tsararano ambony; 11. Ambohimandamina; 12. Sotema; 13. Ambondrona; 14. Amborovy. Cartography base was obtained using OpenStreetMap (OpenStreetMap contributors), kindly provided by Geofabrik under CC-BY 4.0.

**Fig 2:**
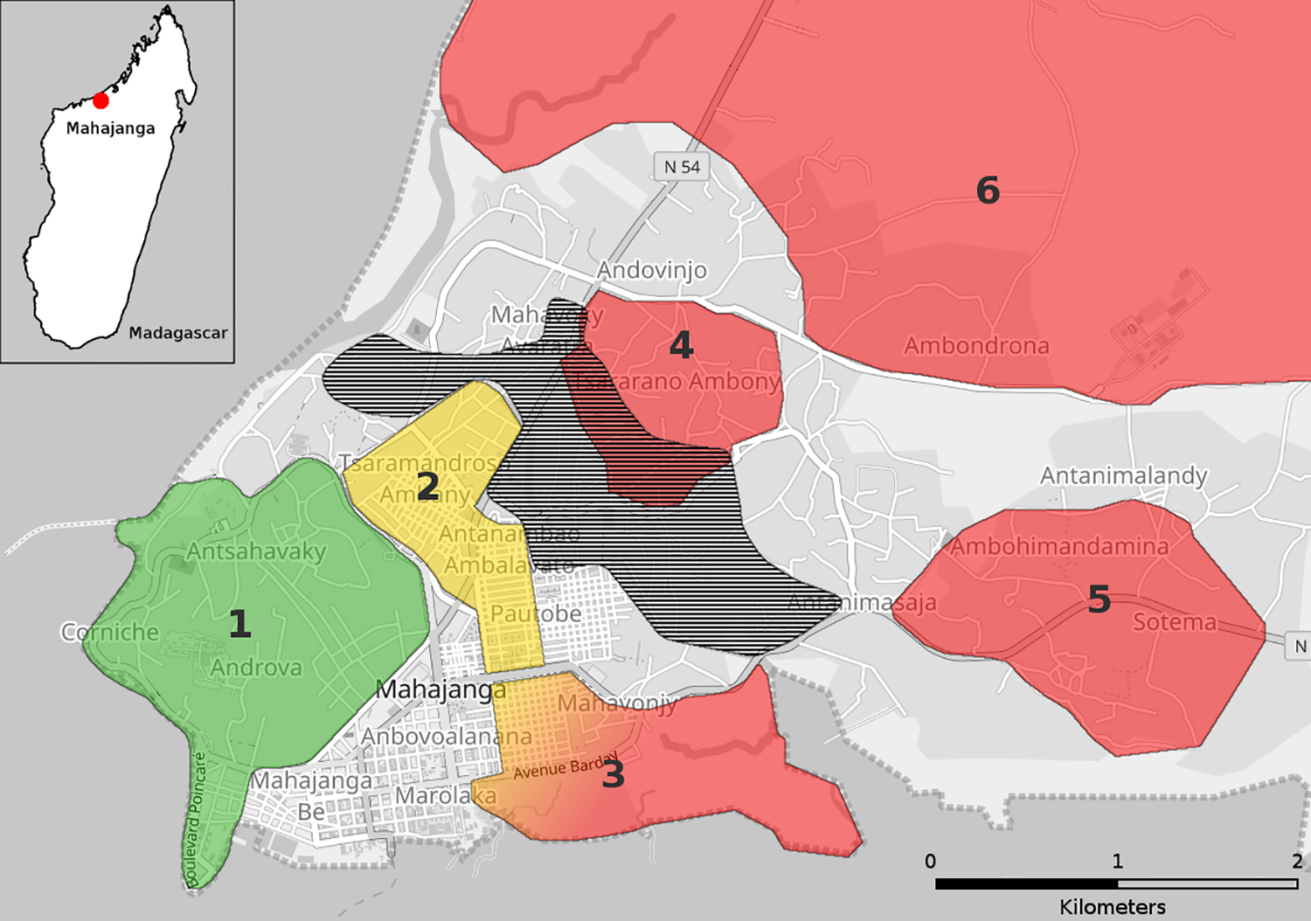
Analysis areas on the map of Mahajanga. Areas are numbered from 1 to 6 according to the description provided in the text, and colored according to their sanitary level: **red** is for poor sanitary level; **yellow** is for medium sanitary level; **green** is for high sanitary level. The flood zone represented (hatched area) corresponds to the area likely to be flooded with water coming from the Metzinger’s canal. Area 3 is likely to be flooded with sea water at high tide (not represented). Cartography base was obtained using OpenStreetMap (OpenStreetMap contributors), kindly provided by Geofabrik under CC-BY 4.0.

The majority (248/265) of the subjects lived in the city of Mahajanga whereas 17 subjects came from the rural area surrounding the city. For analysis, we divided the city of Mahajanga in 6 different areas corresponding to various sanitary and socioeconomic levels, according to the work of F. H. Miandra Rova (2010) and to the map kindly provided by the municipality of Mahajanga (Figs 1 and 2) [23]. Thus, 22 subjects (mean age 32.9±13.7 years, sex ratio 1.00) came from area 1, corresponding to the “Corniche”, “Mangarivotra”, “Androva” and “Antsahavaky” districts, with buried canalizations draining wastewater away, considered good facilities; 41 subjects (mean age 31.0±13.5 years, sex ratio 1.05) came from area 2, corresponding to “Tsaramandroso cité”, “Tsaramandroso ambany” and “Morafeno” districts with open air canalizations; 66 subjects (mean age 38.1±15.1 years, sex ratio 0.53) came from area 3, corresponding to the “Aranta” and “Abattoir” districts which are spontaneous settlements at the Bombetoka bay, likely to be flooded with the tide; 49 subjects (mean age 26.4±10.1 years, sex ratio 0.88) came from area 4, corresponding to the “Tsararano ambony” district which is likely to be flooded with wastewaters coming from the Metzinger’s canal, the principal outlet for wastewater in the city; 58 subjects (mean age 31.5±15.4 years, sex ratio 0.66) came from area 5, corresponding to the “Ambohimandamina” and “Sotema” districts, situated at higher altitude than the rest of the city, without any sanitation facilities; 12 subjects (mean age 27.7±12.3 years, sex ratio 2) came from area 6, corresponding to the rural neighborhoods “Amborovy” and “Ambondrona” with a lower population density. We also included 17 subjects (mean age 42.4±14.9, sex ratio 1.83) from distant rural area (area 7).

### Composition of parasitic fauna

Sixty-one subjects were found to be free of intestinal parasite, with a mean age of 36.1±15.5 years and a sex ratio of 0.62. The 205 subjects harboring at least one parasite formed a group with a mean age of 31.8±14.1 years and a sex ratio of 0,86. The mean age of the infected group was significantly lower than in the parasite free group (Table 2). There was no difference between parasite-free and parasite-infected groups in terms of BMI (respectively 21.88 kg/m^2^ and 22.08 kg/m^2^) and symptoms 30.0% (n = 18/60) *versus* 23.9% (n = 49/205). Moreover, there was no difference between symptomatic and asymptomatic groups in terms of parasite infection rate, with respectively 73.3% and 78.8% subjects carrying at least one parasite.

**Table 2:** Mean age depending on parasite infection.

		Mean age ± std	P value
Parasites	Yes	31.8 ± 14.1	0.043
	No	36.1 ± 15.5	
*Blastocystis*	Yes	31.4 ± 13.9	0.022
	No	35.9 ± 15.4	
*D*. *fragilis*	Yes	24.7 ± 9.4	0.0001
	No	33.6 ± 14.7	
Flagellates	Yes	26.6 ± 11.5	< 0.0001
	No	35.2 ± 14.9	
*G*. *intestinalis*	Yes	23.2 ± 8.0	< 0.0001
	No	33.6 ± 14.7	

A wide panel of intestinal parasites was identified (Table 3). Helminths were present in 7.9% of all the subjects, without any difference between asymptomatic and symptomatic groups. The most prevalent helminth was *Hymenolepis nana* (Table 3), followed by *Schistosoma mansoni*, *Necator americanus* (GenBank accession numbers MH053422-MH053424), *Ancylostoma ceylanicum* (GenBank accession number MH053421), *Trichiuris trichiura* and *Ascaris lumbricoides*.

**Table 3:** Prevalences of parasites species identified among included subjects. “Other amoebas” correspond to parasites which could not be identified by microscopic examination and PCR-based methods.

	Overall Infections and prevalence (%)	Infections in symptomatic subjects and prevalence (%)	Infections in asymptomatic subjects and prevalence (%)	P-value
**PARASITES**	**204 (77.0)**	**48 (71.6)**	**156 (78.8)**	**n. s.**
**Protozoans**	**201 (75.9)**	**46 (68.7)**	**155 (78.3)**	**n. s.**
*Blastocystis* sp.	185 (69.8)	41 (61.2)	144 (72.7)	0.08
Amoebas	94 (35.5)	22 (32.8)	72 (36.4)	n. s.
*E*. *histolytica*	12 (4.5)	3 (4.5)	9 (4.5)	n. s.
*E*. *dispar*	14 (5.3)	5 (7.5)	9 (4.5)	n. s.
*E*. *hartmanii*	21 (7.9)	4 (6.0)	17 (8.6)	n. s.
*E*. *nana*	23 (8.7)	6 (9.0)	17 (8.6)	n. s.
*E*. *coli*	71 (26.8)	16 (23.9)	55 (27.8)	n. s.
*I*. *butschlii*	1 (0.4)	1 (1.5)	0 (0.0)	n. s.
other amoebas	10 (3.8)	2 (3.0)	8 (4.0)	n. s.
Flagellates	78 (29.4)	23 (34.3)	55 (27.8)	n. s.
*D*. *fragilis*	33 (12.5)	8 (11.9)	25 (12.6)	n. s.
*G*. *intestinalis*	21 (7.9)	11 (16.4)	10 (5.1)	0.003
*C*. *mesnili*	27 (10.2)	8 (11.9)	19 (9.6)	n. s.
*P*. *hominis*	6 (2.3)	0 (0.0)	6 (3.0)	n. s.
*E*. *intestinalis*	8 (3.0)	0 (0.0)	8 (4.0)	n. s.
*E*. *hominis*	19 (7.2)	4 (6.0)	15 (7.6)	n. s.
*S*. *similis*	4 (1.5)	2 (3.0)	2 (1.0)	n. s.
Unidentified flagellate	2 (0.8)	0 (0.0)	2 (1.0)	n. s.
*B*. *coli*	1 (0.4)	0 (0.0)	1 (0.5)	n. s.
*Cryptosporidium* sp.	7 (2.6)	0 (0.0)	7 (3.5)	n. s.
**Helminths**	**21 (7.9)**	**7 (10.4)**	**14 (7.1)**	**n. s.**
Cestods	5 (1.9)	2 (3.0)	3 (1.5)	n. s.
*H*. *nana*	5 (1.9)	2 (3.0)	3 (1.5)	n. s.
Nematods	12 (4.5)	3 (4,5)	9 (4,5)	n. s.
*N*. *americanus*	3 (1.1)	1 (1.5)	2 (1.0)	n. s.
*A*. *ceylanicum*	1 (0.4)	0 (0.0)	1 (0.5)	n. s.
Ankylostomidae	4 (1.5)	1 (1.5)	3 (1.5)	n. s.
*T*. *trichiura*	3 (1.1)	1 (1.5)	2 (1.0)	n. s.
*A*. *lumbricoides*	1 (0.4)	0 (0.0)	1 (0.5)	n. s.
Trematods	4 (1.5)	1 (1.5)	3 (1.5)	n. s.
*S*. *mansoni*	4 (1.5)	1 (1.5)	3 (1.5)	n. s.
**Microsporidia**	**12 (4.5)**	**3 (4.5)**	**9 (4.5)**	**n. s.**
*E*. *bieneusi*	6 (2.3)	1 (1.5)	5 (2.5)	n. s.
*E*. *intestinalis*	6 (2.3)	2 (3.0)	4 (2.0)	n. s.

The overall prevalence of protozoa was 75.9% (Table 3), *Blastocystis* sp. being the most prevalent with 69.8%. Taken together, protozoans were not more prevalent in the asymptomatic group (73.7%) or in the symptomatic group (62.7%). The overall prevalence of *Entamoeba* sp. was 34.0%, *Entamoeba coli* being the second most prevalent protozoa of our study (26.4%). The prevalence of *E*. *dispar* and *E*. *histolytica* (identified by PCR) were respectively 4.5% and 5.3%, 7 subjects being infected with both species. *E*. *histolytica* carriage was not significantly associated to intestinal symptoms. For 4 subjects, microscopic examination was in favor of *E*. *histolytica*/*E*. *dispar*, but PCR failed to identify either species. Moreover, in 8 subjects presenting cysts or trophozoites suggestive of *Entamoeba* sp., we were unable to confirm species identification by microscopy and *E*. *histolytica/dispar* PCR. Moreover, all these 12 patients were negative for *E*. *poleckii* and *E*. *moshkowski* PCR (data not shown).

Of all the subjects, 29.4% were found to harbor at least one flagellate (Table 3). The prevalence of these parasites was similar in both symptomatic and asymptomatic groups. In contrast, *Giardia intestinalis* was significantly more prevalent in symptomatic subjects (16.4% *versus* 5.1%, *p =* 0.003). *G*. *intestinalis* carriage was also significantly associated to diarrhea (*p*<0.001), bloating (*p* = 0.036), abdominal pain (*p* = 0.032) and lower BMI (20.16±2.64 kg/m^2^ *versus* 21.83±3.57 kg/m^2^, *p* = 0.012) (S1 Table).

**Fig 3:**
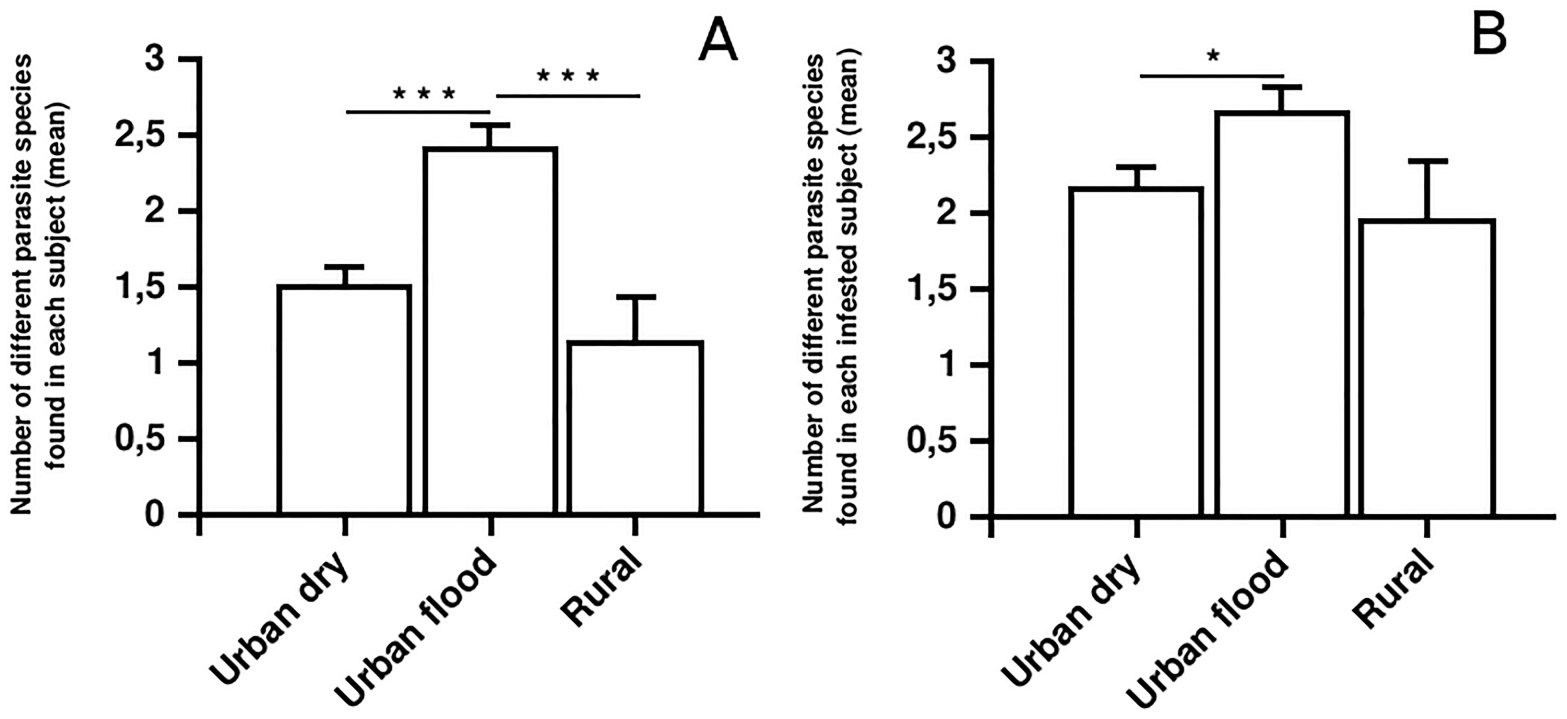
Mean number (and standard error) of parasite species found in (A) all subject or (B) infected subjects. * *p* < 0.05 ** *p* < 0.01 *** *p* < 0.001.

The prevalence of protozoan infections was significantly correlated with the sanitary level of the neighborhood in which the different subjects lived (Table 4). Neighborhoods with a lower sanitary level, especially floody areas, presented higher prevalence of intestinal protozoans in their populations (Table 4). In the rural area (gathering study areas 6 and 7), 58.6% of the subjects were found to harbor at least one parasite *vs*. 79.7% of the subjects living in the urban area (gathering study areas 1 to 5), this difference being statistically significant (*p* = 0.01). Subjects harbored up to 7 different species. Thus, the 187 infected subjects living in the urban area harbored a mean of 2.43 different species while the 17 infected subjects living in the rural area harbored a mean of 2.00 different species(Table 5). The mean number of parasites species isolated from subjects living in urban areas likely to be flooded was significantly higher than in samples from subjects living in other urban areas (Table 5, Fig 3).

**Table 4:** Parasite prevalence according to Mahajanga districts (P-values in S2, S3 and S4 Tables).

	Mahajanga areas*						
	1	2	3	4	5	6	7
**Type of area**	Urban	Urban	Urban	Urban	Urban	Semi-rural	Rural
Sanitary level**	High	Medium	Low***	Low***	Low	Low	Unknown
**Parasites**	68.18	63.41	90.91	89.80	74.14	58.33	58.82
1 parasite	31.82	31.71	33.34	28.57	27.59	16.67	47.06
2 parasites	18.18	19.51	18.18	12.24	13.79	16.67	11.76
≥ 3 parasites	18.18	12.20	39.39	48.98	32.76	25.00	0
***Blastocystis***	59.09	56.10	87.88	85.71	62.07	58.33	35.29
***D*. *fragilis***	22.73	7.32	3.03	18.37	8.62	8.33	0
**Amoebas**	13.64	14.63	51.51	48.98	34.48	41.67	11.76
**Flagellates (without *D*. *fragilis*)**	9.09	12.20	19.70	40.82	27.59	16.67	5.88
*G*. *intestinalis*	4.55	2.44	0.00	16.33	15.52	16.67	5.88

* 1: Corniche, Mangarivotra, Androva and Antsahavaky districts; 2: Tsaramandroso cite, Tsaramandroso ambany and Morafeno districts; 3: Aranta and Abattoir districts; 4: Tsararano ambony district; 5: Ambohimandamina and Sotema districts; 6: Amborovy and Ambondrona; 7 distant rural1 area.

** according to Mahajanga municipal register.

*** floody area.

**Table 5:** Average number of infesting parasite species in each study area (standard deviation in brackets).

Area	1	2	3	4	5	6	7
Mean numbers of parasite species in each subject	1.32 [0.03–2.61]	1.20 [0.00–2.67]	2.32 [0.56–4.08]	2.63 [0.79–4.47]	1.86 [0.13–3.59]	1.75 [0.00–3.97]	0.71 [0.02–1.40]
Mean numbers of parasite species in infected subjects	1.93 [0.83–3.03]	1.96 [0.53–3.39]	2.55 [0.88–4.22]	2.93 [1.23–4.63]	2.51 [0.96–4.06]	3.00 [0.84–5.16]	1.20 [0.78–1.62]

### Distribution of *Blastocystis* sp. subtypes

We were able to subtype 158 of the 171 *Blastocystis* sp. PCR positive samples, 13 samples presenting overlapped chromatograms suggesting mixed-ST infections. Three different STs were found in our study (Table 6). ST1 was the most prevalent subtype (46.8%), followed by ST3 (24.6%) and ST2 (21.1%). There were no correlation between ST distribution and epidemiological features of included subjects.

**Table 6:** *Blastocystis* subtype distribution in Africa.

Country	Total samples	ST1	ST2	ST3	ST4	ST6	ST7	Non typable	Mixed infection	Reference
Egypt	44	18.2	0.0	54.5	0.0	18.2	9.1	0.0	0.0	Hussein et al., 2008
Egypt	21	19.0	19.0	61.9	0.0	0.0	0.0	0.0	0.0	Souppart et al., 2010
Egypt	33	0.0	0.0	100.0	0.0	0.0	0.0	0.0	0.0	Hameed et al., 2011
Egypt	110	13.6	0.0	44.5	0.0	30.0	11.8	0.0	0.0	Fouad et al., 2011
Egypt	71	16.9	0.0	83.1	0.0	0.0	0.0	0.0	0.0	El Deeb et al., 2013
Libya	42	54.8	26.2	19.0	0.0	0.0	0.0	0.0	0.0	Abdulsalam et al., 2013
Libya	38	50.0	7.9	39.5	0.0	0.0	2.6	0.0	0.0	Alfellani et al., 2013
Tunisia	61	30.0	16.0	51.0	1.6	0.0	1.6	0.0	0.0	Ben Abda et al., 2017
Senegal	100	28.0	20.0	50.0	2.0	0.0	0.0	0.0	0.0	El Safadi et al., 2014
Liberia	25	28.0	28.0	32.0	12.0	0.0	0.0	0.0	0.0	Alfellani et al., 2013
Ivory Coast	110	50.0	22.0	28.0	0.0	0.0	0.0	0.0	0.0	D’Alfonso et al., 2017
Nigeria	22	45.5	0.0	40.9	13.6	0.0	0.0	0.0	0.0	Alfellani et al., 2013
Nigeria	127	40.2	33.1	26.0	0.0	0.0	0.7	0.0	0.0	Poulsen et al., 2017
Tanzania	106	34.0	26.4	25.5	0.0	0.0	0.9	13.2	0.0	Forsell et al., 2016
*Madagascar*	*171*	*46*.*8*	*21*.*1*	*24*.*6*	*0*.*0*	*0*.*0*	*0*.*0*	*0*.*0*	*7*.*5*	*Present study*
**Total**	**1081**	**33.9**	**17.1**	**40.0**	**0.8**	**3.8**	**1.9**	**1.3**	**1.2**	

### Distribution of *D*. *fragilis* genotypes

**Fig 4:**
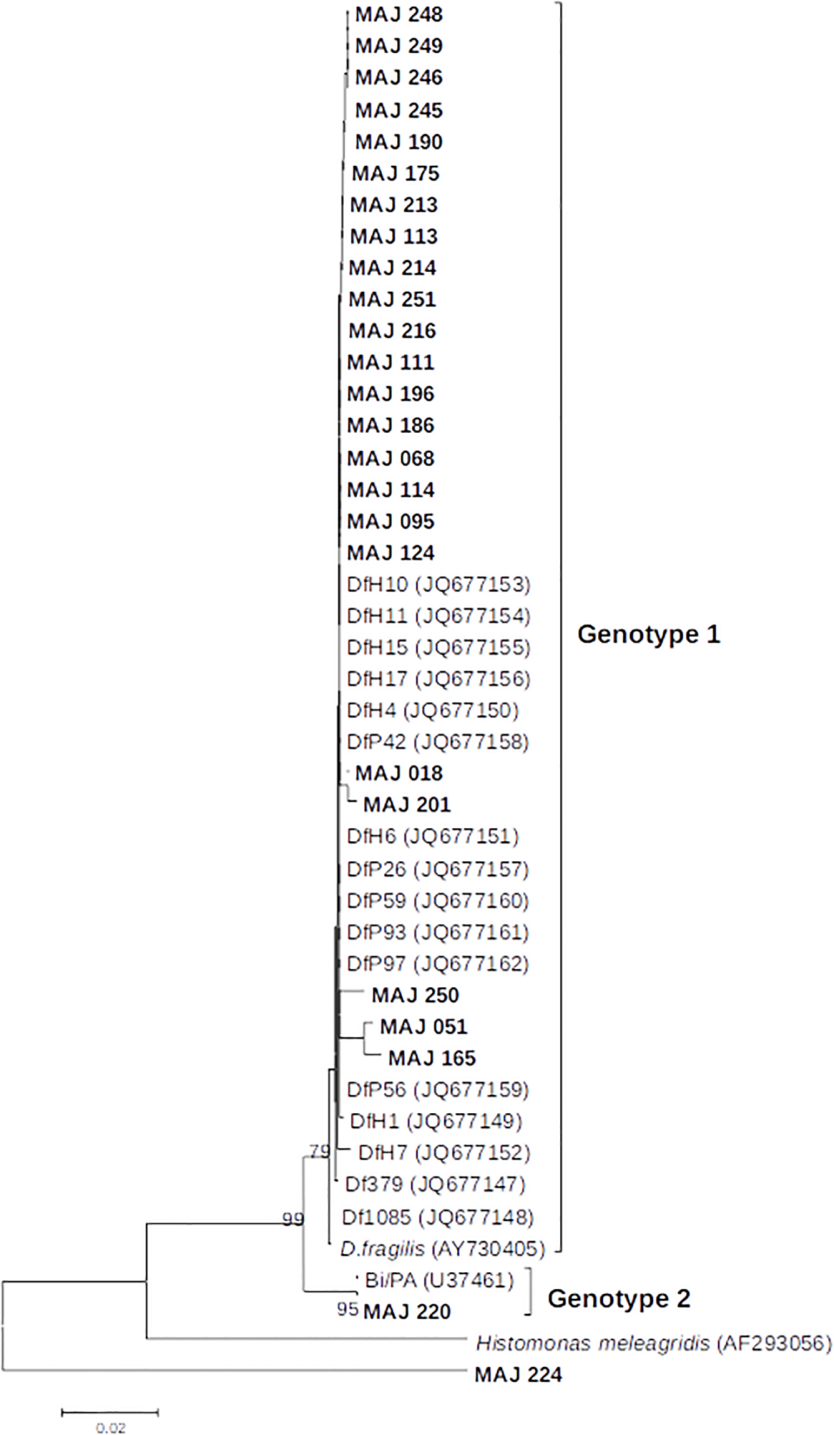
Molecular phylogenetic analysis of *D*. *fragilis* isolates based on ITS sequences. The evolutionary history was inferred by using the Maximum Likelihood method based on the Tamura 3-parameter model (1000 replicates) using MEGA6. Only bootstrap values higher than 70% are shown. Reference sequences from genotype 1 and 2 were included in the analysis. Isolates from the present study are highlighted in bold. Isolate MAJ 224 correspond to the *Simplicimonas similis* positive stool sample.

There were 33 positive stool samples using the *D*. *fragilis* real-time PCR (Table 3). We were able to genotype 25/33 positive samples for *D*. *fragilis*. Sequence analyses showed genotype 1 in 23 subjects and genotype 2 in one subject. Furthermore, one isolate (MAJ 224) that did not cluster the *Dientamoeba* genus but with another tritrichomonad species, *Simplicimonas similis* (Fig 4).

### Simplicimonas similis

**Fig 5:**
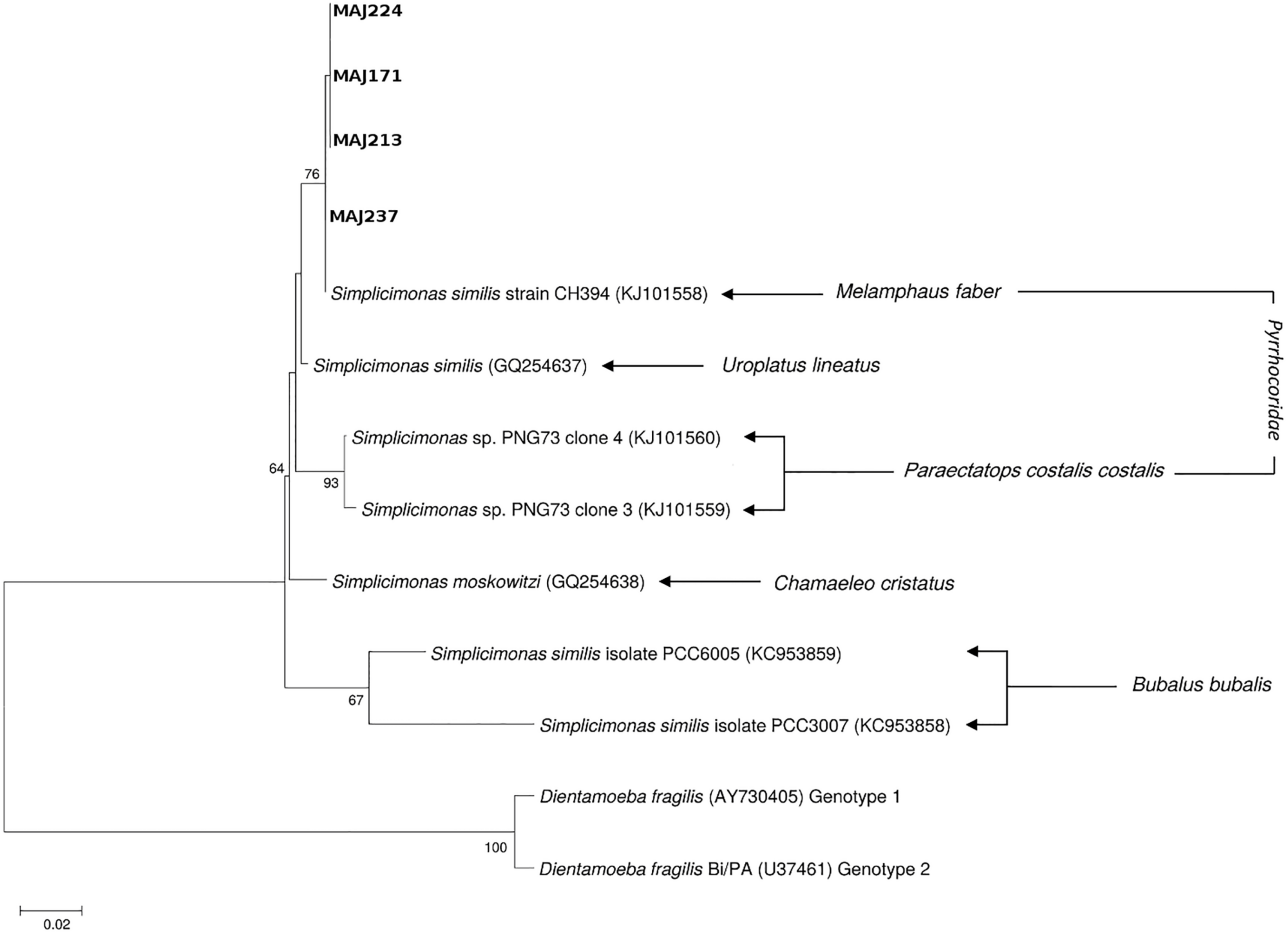
Molecular phylogenetic analysis of the four *S*. *similis* strains isolated in our study based on 18S rRNA sequences. The evolutionary history was inferred by using the Maximum Likelihood method based on the Tamura 3-parameter model (1000 replicates) using MEGA6. Only bootstrap values higher than 70% are shown. The strains found in human stool (MAJ 224, MAJ171, MAJ 213, MAJ 237) are similar to each other and very similar to the CH394 strain found in *Melampheus faber*.

Since we found one sample bearing *S*. *similis* DNA, we developed a specific real-time PCR assay and screened all included subjects in order to increase the detectability of *S*. *similis*. PCR products of each positive sample were subsequently sequenced to confirm the presence of the parasite. With this technique, 3 additional positive subjects were identified. Two of the four subjects, three men and one woman, aged between 21 and 49 years, complained about symptoms, and all were infected with other parasites. Three subjects lived in low sanitary level neighborhoods (2 in area 2 and 1 in area 4) and 1 lived in the highest sanitary level quarter of area 1. The four isolates (GenBank accession numbers MH053425-MH053428) presented a high homology with each other and with the *S*. *similis* strain CH394 (Fig 5).

One of the carriers of *S*. *similis* was re-sampled one year later as well as two members of his family and his zebu (*Bos taurus indicus*). The first subject was positive again, as well as one of the two family members (GenBank accession number MH057079) and the zebu (GenBank accession number MH057080). All isolates presented a high homology with the CH394 strain (Fig 5).

## Discussion

### Prevalence and evolution over time

We studied stool samples from a consistent number of subjects with a relatively low average age, coherent with Malagasy demographic specificities (http://www.who.int/countries/mdg/en/). In our study, 77.0% of all subjects were carrying at least one intestinal parasite. Without considering *Blastocystis* sp., the prevalence was 50.6%. This prevalence is high and particularly relevant when compared with the results of a similar study conducted in the same city in 2003 [8]. In this work, the proportion of subjects carrying at least one intestinal parasite was 67.6%. This result is comparable with results of other prevalence studies conducted in Africa, including Nigeria and Ethiopia, with prevalence between 83% and 97% [24–26]. The vast majority of parasites found in our samples were protozoans, whereas the prevalence of helminths was relatively low (7.9% of all the subjects representing 5.1% of all the parasites found). Helminths were mostly *H*. *nana* and *S*. *mansoni*. This result contrasts with the 2003 study in which 23.4% of the subjects were carrying helminths. This decrease of helminths prevalences over time results from mass drug administration campaigns, which started in 2002 using mebendazole and albendazole. The prevalence of cestods was low, which was already the case in the 2003 study [8]. Regarding the prevalence of protozoa, the relative abundance of the different species was quite similar between our study and the 2003 study, even though *Blastocystis* sp. prevalence was not provided by Buchy *et al*. in 2003 [8]. However, three species were more abundant in our study, *D*. *fragilis* (9.4% vs. 1.7%), *Chilomastix mesnili* (10.2% vs. 3.2%) and *Enteromonas hominis* (7.2% vs. 1.7%). This may be explained by the difference in diagnostic methods used: we used protozoa culture and a PCR technique for *D*. *fragilis*, which was not the case in 2003. However, in the present study, we did not include children under 18 years old whereas the 2003 study included children from the age of 1 year, making these differences difficult to interpret.

The majority of intestinal parasites are acquired *via* fecal-oral transmission. Thus, the fact that an important proportion of the subjects have no access to sanitary infrastructures may explain this result. Indeed, Madagascar is one of the poorest countries in the world with a low Human Development Index (HDI) [27]. Furthermore, in 2010, in the Mahajanga area, the incidence of poverty was 71.6% [27]. In the Boeny district, where Mahajanga is situated, less than 20% of the population declared using at least latrines [27]. There was a strong correlation between different neighborhoods of different sanitary levels and the prevalence of intestinal parasites. Indeed, we found the highest prevalences in the neighborhoods with the lowest sanitary level, like “Abattoir”, “Aranta” or “Tsararano ambony”. Such a correlation had already been described in Ethiopia and Nigeria [24,25]. However, in our study, these differences were inconstant and the prevalence in the “Corniche” neighborhood with high sanitary level was not significantly lower than in the neighborhoods with medium/low sanitary levels “Amborovy” and “Ambondrona”. Moreover, people living in regularly flooded and densely populated areas were more subjected to intestinal parasitosis. Indeed, the “Amborovy” and “Ambondrona” rural areas are less densely populated than “Abattoir”, “Aranta” and “Tsararano”. Thus, intestinal parasites seems to expand more efficiently in highly dense populations, particularly among mammals populations [28,29]. This hypothesis is corroborated by the low prevalence of *Blastocystis* sp. in the strictly rural population (coming from outside the city of Mahajanga), as well as the difference between the mean species numbers found in positive subjects.

### Clinical manifestations and pathogenicity

Whereas *G*. *intestinalis* was more prevalent in symptomatic group, overall protozoa infections were more frequent in asymptomatic subjects. Furthermore, the number of different species found in positive asymptomatic and symptomatic infected subjects was similar (2.37 *vs*. 2.49 respectively). This suggests that, except of *G*. *intestinalis*, most intestinal parasites are not pathogenic enough to induce acute gastro-intestinal symptoms. It is also possible that some protozoa may be a part of the “normal” eukaryotic intestinal microfauna in tropical areas. However, the long term consequences of a contamination with intestinal parasites are not predictable in such a “snapshot” study. Indeed, it is possible that long term interactions between the host and intestinal parasites are, in part, responsible for infraclinical symptoms such as malabsorption or immunity impairment, and *in fine*, growth retardation, limitation of the cognitive development or reduction of the life expectancy.

The pathogenicity of the two emerging protozoans *Blastocystis* sp. and *D*. *fragilis* has been widely discussed in the past few years. In our study, *Blastocystis* sp. was more prevalent in asymptomatic subjects and we did not find any correlation between subtypes and symptoms. However, *Blastocystis* sp. was widely associated with other intestinal parasites, which makes our interpretations difficult. In our study, 9.4% of subjects were infected with *D*. *fragilis*. Other studies found prevalences ranging from 0.2% to 82.9% [30]. In the majority of these studies, diagnosis was made using microscopic examinations which is not as sensitive as molecular tools. Only four studies used molecular techniques, finding prevalences of around 5% in Australia [31,32], 32% in the Netherlands [33] and 21.4% in Italy [34]. Our results suggest that *D*. *fragilis* is not a “tropical” parasite, but further studies in Africa are required. Moreover, all *D*. *fragilis* carriers were also positive for *Blastocystis* sp., and 28% complained of symptoms.

### *Blastocystis* sp. subtypes

Until now, 10 different subtypes (i.e ST1-ST9 and ST12) of *Blastocystis* are known to infect human, ST3 being the most prevalent, followed by ST1, ST2 and ST4 [35,36]. In our study, among the 158 subtyped isolates, only three different STs were identified, ST1 to ST3, ST1 being the most common. Similar results had already been found in Africa (Table 6), but such a repartition of STs is not the most frequent situation worldwide or in Africa where ST3 is often the most prevalent ST [22]. The absence of ST4 was congruent with previous studies performed in Africa, this ST being more prevalent in Europe. The relatively low subtype diversity observed in our study may be linked to the insularity of Madagascar.

### *D*. *fragilis* genotypes

Only two different genotypes of *D*. *fragilis*, G1 and G2, have been described [30]. This low genetic diversity has been recently confirmed by multilocus sequence typing of isolates from different regions of the world [37]. This study showed the worldwide predominance of G1 and the relative rarity of G2. A recent study in Brazil found only G1 in 19 isolates [38] but there is currently no data regarding to Africa. In our cohort, one of the 23 positive subjects, an asymptomatic man living in the poor sanitary level neighborhood “Aranta”, was infected with G2. This shows at least the circulation of this rare genotype on the island. Studies on a larger scale may be interesting to assess the circulation of G2 in Madagascar or other African countries to determine whether this genotype is more frequent between the tropics than in the temperate area and if it is associated with poor sanitary conditions. Furthermore, it may be interesting to compare pathogenicity between these two genotypes.

### First description of *Simplicimonas similis* DNA in human stool

We have, incidentally, identified the presence of *Simplicimonas similis* DNA in the stool of 4 subjects. This parasite take place within the superclass of parabasalids, along with the tritrichomonads (including *D*. *fragilis*). *S*. *similis* is a small flagellate which was first observed in the stool of a the gecko species *Uroplatus lineatus*, endemic to Madagascar, and described in 2010 by Cepicka *et al*. [39]. *Simplicimonas* sp. have since been described in Asian buffalo (*Bubalus bubalis*) [40] and in two species of bugs, *Paraectatops costalis costalis* and *Melampheus faber* [41]. A flagellate described as *Simplicimonas* sp.-like had been described in chicken in 2011 [42]. It has to be mentioned that we only detected DNA from *S*. *similis*, while we were unable to detect it by microscopy. Consequently, in view of the similarity between our strains and the one described in bugs, we cannot rule out an alimentary consumption of insects by the study subjects, to be responsible for these findings. However, one of the positive subjects was still positive one year later, suggesting either the possibility of a stable infection, re-infection, or continued exposure to *S*. *similis* in the study subjects diet. We also found positive results for one of his family members and his Zebu. Further studies are now required to isolate living parasite from human stool in order to confirm or refute that humans are natural hosts of *S*. *similis*.

### Conclusion

Our work provides a snapshot of intestinal parasitic fauna in a Malagasy cohort 12 years after a first report in the same city, Mahajanga. Changes in living and sanitary conditions of developing countries as well as public health, hygiene-dietetic or medical interventions aimed at limiting the transmission of these infections. Thus, in Mahajanga, the prevalence of helminths infections has critically decreased over the past twelve years, highlighting the efficiency of anti-helminthic campaigns. However, the prevalence of protozoan infections is stable or, for a few species, has increased over the same period. Thus, intestinal parasites are highly prevalent in poorest districts of Mahajanga, *Blastocystis* sp. being the most prevalent. This suggests an increase in fecal contamination of the environment, correlated to the population growth.

Molecular analyses of a few protozoan species, namely *D*. *fragilis* and *Blastocystis* sp., provide informations regarding to the specific epidemiology of these micro-organisms. Thus, our study confirm that the prevalence of *D*. *fragilis* is probably lower in tropical areas than in western countries, but genotype 1 remains highly predominant. On the other hand, the prevalence of *Blastocystis* sp. is higher in developing countries such as Madagascar, but only common subtypes (i.e. ST1, ST2 and ST3) were identified in our study.

Finally, we were able to detect, for the first time in human samples, DNA of a recently described protozoa, *S*. *similis*. However, more studies are required to assess the life cycle and the potential pathogenicity of this eukaryote, and whether or not it plays a role in the human intestinal microfauna.

Data from our study provide valuable key for sanitation programs and prevention of fecal-related infectious diseases.

## Supporting information

S1 Table Symptoms in *G*. *intestinalis* carriers *vs*. non-carriers. (DOCX)

S2 Table P-values of the differences of prevalence between Mahajanga areas for parasites (all together). (DOCX)

S3 Table P-values of the differences of prevalence between Mahajanga areas for *Blastocystis* sp. (DOCX)

S4 Table P-values of the differences of prevalence between Mahajanga areas for flagellates (all together). (DOCX)

S5 Table Results for parasites for each subject included, using microscopy or molecular biology. When applicable, Ct are indicated in brackets.
(PDF)
