# Supplementary material for: Human intestinal parasites in Mahajanga, Madagascar: The kingdom of the protozoa
Source: PLoS One. 2018 Oct 10;13(10):e0204576. doi: 10.1371/journal.pone.0204576 (PMC6179227; doi:10.1371/journal.pone.0204576)
Supplement: S1 Table — (DOCX) [file pone.0204576.s001.docx]

**S1 Table – Symptoms in *G. intestinalis* carriers *vs*. non-carriers.**

|  | **Yes (n=21)** | **No (n=244)** | **p-value** |
| --- | --- | --- | --- |
| Symptoms (overall) | 11 (52.4%) | 57 (23.4%) | **0.004** |
| Diarrhea | 8 (38.1%) | 23 (9.4%) | **<0.001** |
| Bloating | 5 (23.8%) | 20 (8.2%) | **0.036** |
| Abdominal pain | 7 (33.3%) | 36 (14.8%) | **0.032** |
| Constipation | 1 (4.8%) | 8 (3.3%) | 0.53 |
| Arthralgia | 1 (4.8%) | 9 (3.7%) | 0.56 |
| BMI | 20.16±2.64 | 21.83±3.57 | **0.012** |
