# Supplementary material for: Human intestinal parasites in Mahajanga, Madagascar: The kingdom of the protozoa
Source: PLoS One. 2018 Oct 10;13(10):e0204576. doi: 10.1371/journal.pone.0204576 (PMC6179227; doi:10.1371/journal.pone.0204576)
Supplement: S2 Table — (DOCX) [file pone.0204576.s002.docx]

**S2 Table – P-values of the differences of prevalence between Mahajanga areas for parasites (all together).**

| **Area*** | **1**  **(n = 22)** | **2**  **(n = 41)** | **3**  **(n = 66)** | **4**  **(n = 49)** | **5**  **(n = 58)** | **6**  **(n = 12)** | **7**  **(n = 17)** |
| --- | --- | --- | --- | --- | --- | --- | --- |
| **Type of area** | Urban | Urban | Urban | Urban | Urban | Semi-rural | Rural |
| **Sanitary level**** | High | Medium | Low^***^ | Low^***^ | Low | Low | Unknown |
| **1** |  |  |  |  |  |  |  |
| **2** | 0.82 |  |  |  |  |  |  |
| **3** | **0.002** | **<0.0001** |  |  |  |  |  |
| **4** | 0.051 | **0.0085** | 0.23 |  |  |  |  |
| **5** | 0.36 | 0.13 | **0.0085** | 0.19 |  |  |  |
| **6** | 0.93 | 0.89 | **0.0061** | 0.085 | 0.40 |  |  |
| **7** | 0.43 | 0.53 | **<0.0001** | **0.0058** | 0.072 | 0.55 |  |

* 1: Corniche, Mangarivotra, Androva and Ambalaronby districts; 2: Tsaramandroso cite, Tsaramandroso ambany and Morafeno districts; 3: Aranta and Abattoir districts; 4: Tsararano ambony district; 5: Ambohimandamina and Sotema districts; 6: Amborovy and Ambondrona; 7 distant rural1 area.

** according to Mahajanga municipal register.

*** floody area.
