# Supplementary material for: Human intestinal parasites in Mahajanga, Madagascar: The kingdom of the protozoa
Source: PLoS One. 2018 Oct 10;13(10):e0204576. doi: 10.1371/journal.pone.0204576 (PMC6179227; doi:10.1371/journal.pone.0204576)
Supplement: S3 Table — (DOCX) [file pone.0204576.s003.docx]

**S3 Table – P-values of the differences of prevalence between Mahajanga areas for *Blastocystis* sp.**

| **Area*** | **1**  **(n = 22)** | **2**  **(n = 41)** | **3**  **(n = 66)** | **4**  **(n = 49)** | **5**  **(n = 58)** | **6**  **(n = 12)** | **7**  **(n = 17)** |
| --- | --- | --- | --- | --- | --- | --- | --- |
| **Type of area** | Urban | Urban | Urban | Urban | Urban | Semi-rural | Rural |
| **Sanitary level**** | High | Medium | Low^***^ | Low^***^ | Low | Low | Unknown |
| **1** |  |  |  |  |  |  |  |
| **2** | 0.60 |  |  |  |  |  |  |
| **3** | **0.017** | **0.00014** |  |  |  |  |  |
| **4** | 0.074 | **0.0015** | 0.80 |  |  |  |  |
| **5** | 0.81 | 0.55 | **0.0003** | **0.004** |  |  |  |
| **6** | 0.72 | 1 | **0.013** | 0.055 | 0.76 |  |  |
| **7** | **0.049** | 0.089 | **<0.0001** | **<0.0001** | **0.028** | 0.24 |  |

* 1: Corniche, Mangarivotra, Androva and Ambalaronby districts; 2: Tsaramandroso cite, Tsaramandroso ambany and Morafeno districts; 3: Aranta and Abattoir districts; 4: Tsararano ambony district; 5: Ambohimandamina and Sotema districts; 6: Amborovy and Ambondrona; 7 distant rural1 area.

** according to Mahajanga municipal register.

*** floody area.
