# Supplementary material for: Human intestinal parasites in Mahajanga, Madagascar: The kingdom of the protozoa
Source: PLoS One. 2018 Oct 10;13(10):e0204576. doi: 10.1371/journal.pone.0204576 (PMC6179227; doi:10.1371/journal.pone.0204576)
Supplement: S5 Table — When applicable, Ct are indicated in brackets. (PDF) [file pone.0204576.s005.pdf]

S5 Table – Results for parasites for each subject included, using microscopy or molecular biology. When applicable, Ct are indicated in brackets.

Subject	Area	Microscopy				Molecular biology								
		**Direct examination**	**MIF**	**Faust**	**Culture**	**qPCR *D. fragilis***	**qPCR *Simplicimonas* sp.**	**qPCR *Blastocystis* sp.**	**qPCR *E. bieneusi***	**qPCR *E. intestinalis***	**qPCR *Cryptosporidium* sp.**	**PCR *E. histolytica/E . dispar***	**PCR *N. americanus***	**PCR *A. ceylanicum***
DF001	VII	NEG	*- E. coli*	*- S. mansonii (eggs)*	NEG	NEG	NEG	NEG	NEG	NEG	NEG	-	-	-
DF002	II	NEG	NEG	NEG	NEG	NEG	NEG	NEG	NEG	NEG	NEG	-	-	-
DF003	II	NEG	*- E. hartmanni*	NEG	*- Amoeba*	NEG	NEG	NEG	NEG	NEG	NEG	-	-	-
DF004	IV	*- Blastocystis sp.*	*- Blastocystis sp.*	NEG	*- Blastocystis sp.*	NEG	NEG	POS<br>(> 40.00)	NEG	NEG	NEG	-	-	-
DF005	VII	*- G. intestinalis*	*- G. intestinalis*	*- S. mansonii (eggs)*	NEG	NEG	NEG	NEG	NEG	NEG	NEG	-	-	-
DF006	III	*- Blastocystis sp.*	*- D. fragilis*<br>*- Blastocystis sp.*<br>*- E. coli*<br>*- E. histolytica/dispar*<br>*- E. nana*<br>*- E. hartmanni*	NEG	*- D. fragilis*<br>*- Blastocystis sp.*<br>*- E. coli*<br>*- E. nana*<br>*- E. hartmanni*	NEG	NEG	POS<br>(28.04)	NEG	NEG	NEG	POS	-	-
DF007	V	NEG	NEG	NEG	NEG	NEG	NEG	NEG	NEG	NEG	NEG	-	-	-
DF008	II	NEG	NEG	NEG	NEG	NEG	NEG	NEG	NEG	NEG	NEG	-	-	-
DF009	III	*- Blastocystis sp.*	*- Blastocystis sp.*<br>*- E. coli*<br>*- E. histolytica/dispar*	NEG	*- Blastocystis sp.*<br>*- Amoeba*	NEG	NEG	POS<br>(20.91)	NEG	NEG	NEG	POS	-	-
DF010	II	NEG	*- Blastocystis sp.*	NEG	*- Blastocystis sp.*	NEG	NEG	POS<br>(34.92)	NEG	NEG	NEG	-	-	-
DF011	II	NEG	NEG	NEG	NEG	NEG	NEG	POS<br>(28.46)	NEG	NEG	NEG	-	-	-
DF012	VII	NEG	*- Blastocystis sp.*	NEG	*- Blastocystis sp.*	NEG	NEG	POS<br>(22.58)	NEG	NEG	NEG	-	-	-
DF013	I	NEG	NEG	NEG	NEG	NEG	NEG	NEG	NEG	NEG	NEG	-	-	-

DF015	I	*- D. fragilis*<br>*- Blastocystis sp.*<br>*- E. coli*<br>*- E. hartmanni*	*- D. fragilis*<br>*- Blastocystis sp.*<br>*- E. coli*<br>*- E. hartmanni*	NEG	*- D. fragilis*<br>*- Blastocystis sp.*<br>*- E. coli*<br>*- E. hartmanni*	POS<br>(26.89)	NEG	POS<br>(31.24)	NEG	NEG	NEG	-	-	-
DF016	I	*- Blastocystis sp.*	*- Blastocystis sp.*	NEG	*- Blastocystis sp.*	NEG	NEG	POS<br>(26.18)	NEG	NEG	NEG	-	-	-
DF017	I	NEG	NEG	NEG	NEG	NEG	NEG	NEG	NEG	NEG	NEG	-	-	-
DF018	I	*- Blastocystis sp.*	*- Blastocystis sp.*	NEG	*- Blastocystis sp.*	POS<br>(28.54)	NEG	POS<br>(24.84)	NEG	NEG	NEG	-	-	-
DF019	I	*- G. intestinalis*<br>*- Amoeba*	*- G. intestinalis*	NEG	*- Blastocystis sp.*	NEG	NEG	NEG	NEG	NEG	NEG	-	-	-
DF020	I	NEG	NEG	NEG	*- Blastocystis sp.*	NEG	NEG	POS<br>(25.79)	NEG	NEG	NEG	-	-	-
DF021	IV	*- Blastocystis sp.*	NEG	NEG	NEG	NEG	NEG	POS<br>(38.32)	NEG	NEG	NEG	-	-	-
DF022	IV	NEG	NEG	NEG	NEG	NEG	NEG	NEG	NEG	NEG	NEG	-	-	-
DF024	VII	*- Blastocystis sp.*	*- Blastocystis sp.*	NEG	*- Blastocystis sp.*<br>*- D. fragilis*	NEG	NEG	POS<br>(32.65)	NEG	NEG	NEG	-	-	-
DF025	IV	*- E. coli*	*- E. coli*	NEG	*- Blastocystis sp.*	NEG	NEG	POS<br>(25.23)	NEG	NEG	NEG	-	-	-
DF026	II	*- Blastocystis sp.*	NEG	NEG	NEG	NEG	NEG	POS<br>(> 40.00)	NEG	NEG	NEG	-	-	-
DF027	VII	NEG	*- Blastocystis sp.*	NEG	*- Blastocystis sp.*	NEG	NEG	POS<br>(34.30)	NEG	NEG	NEG	-	-	-
DF028	II	*- Blastocystis sp.*<br>*- Flagellate*	*- Blastocystis sp.*	NEG	*- Blastocystis sp.*	NEG	NEG	POS<br>(30.86)	NEG	NEG	NEG	-	-	-
DF030	VII	*- E. coli*	*- E. coli*	NEG	*- E. coli*	NEG	NEG	NEG	NEG	NEG	NEG	-	-	-
DF031	I	NEG	NEG	NEG	NEG	NEG	NEG	NEG	NEG	NEG	NEG	-	-	-
DF032	IV	NEG	NEG	NEG	NEG	NEG	NEG	NEG	NEG	NEG	NEG	-	-	-
DF033	I	NEG	NEG	NEG	NEG	NEG	NEG	NEG	NEG	NEG	NEG	-	-	-
DF034	II	NEG	NEG	NEG	NEG	NEG	NEG	POS<br>(32.54)	NEG	NEG	NEG	-	-	-
DF035	II	NEG	NEG	NEG	NEG	NEG	NEG	NEG	NEG	NEG	NEG	-	-	-

DF036	II	NEG	NEG	NEG	NEG	NEG	NEG	NEG	NEG	NEG	NEG	-	-	-
DF037	I	- *Blastocystis sp.*	- *Blastocystis sp.*	NEG	- *Blastocystis sp.*	NEG	NEG	POS<br>(22.48)	NEG	NEG	NEG	-	-	-
DF038	V	NEG	- *Blastocystis sp.*	NEG	NEG	NEG	NEG	POS<br>(27.45)	NEG	NEG	NEG	-	-	-
DF039	VII	NEG	NEG	NEG	NEG	NEG	NEG	NEG	NEG	NEG	NEG	-	-	-
DF040	II	NEG	NEG	NEG	NEG	NEG	NEG	NEG	NEG	NEG	NEG	-	-	-
DF041	V	NEG	NEG	NEG	NEG	NEG	NEG	NEG	NEG	NEG	NEG	-	-	-
DF042	IV	- *Blastocystis sp.*	- *Blastocystis sp.*	NEG	- *Blastocystis sp.*	NEG	NEG	POS<br>(30.49)	NEG	NEG	NEG	-	-	-
DF043	IV	NEG	- *Blastocystis sp.*	NEG	- *Blastocystis sp.*	NEG	NEG	POS<br>(36.45)	NEG	NEG	NEG	-	-	-
DF044	II	NEG	- *Blastocystis sp.*	- *S. mansonii (eggs)*	NEG	NEG	NEG	POS<br>(29.99)	NEG	NEG	NEG	-	-	-
DF045	VI	- *E. coli*	- *Blastocystis sp.*<br>- *E. coli*	NEG	- *Blastocystis sp.*<br>- *E. coli*	NEG	NEG	POS<br>(26.64)	NEG	NEG	NEG	-	-	-
DF046	VI	- *Blastocystis sp.*	- *Blastocystis sp.*	NEG	- *Blastocystis sp.*	NEG	NEG	POS<br>(28.22)	NEG	NEG	NEG	-	-	-
DF047	VI	NEG	NEG	NEG	NEG	NEG	NEG	NEG	NEG	NEG	NEG	-	-	-
DF048	VII	- *Blastocystis sp.*	- *Blastocystis sp.*	NEG	- *Blastocystis sp.*	NEG	NEG	POS<br>(22.52)	NEG	NEG	NEG	-	-	-
DF049	VII	NEG	NEG	NEG	NEG	NEG	NEG	POS<br>(34.21)	NEG	NEG	NEG	-	-	-
DF050	II	NEG	NEG	NEG	NEG	NEG	NEG	POS<br>(25.60)	NEG	NEG	NEG	-	-	-
DF051	II	NEG	- *Blastocystis sp.*	NEG	- *Blastocystis sp.*	POS<br>(27.43)	NEG	POS<br>(26.58)	NEG	NEG	NEG	-	-	-
DF052	II	NEG	- *Blastocystis sp.*	NEG	- *Blastocystis sp.*	NEG	NEG	POS<br>(24.07)	NEG	NEG	NEG	-	-	-
DF053	II	NEG	NEG	NEG	NEG	NEG	NEG	NEG	NEG	NEG	NEG	-	-	-
DF054	III	- *D. fragilis*	- *D. fragilis*	NEG	- *D. fragilis*	POS<br>(28.03)	NEG	NEG	NEG	POS<br>(37.88)	NEG	-	-	-
DF055	II	NEG	NEG	NEG	NEG	NEG	NEG	NEG	NEG	NEG	NEG	-	-	-
DF056	V	- *Blastocystis sp.*	- *Blastocystis sp.*	NEG	NEG	NEG	NEG	POS<br>(37.68)	NEG	NEG	NEG	-	-	-

DF057	V	*- E. coli*	*- E. coli*<br>*- Blastocystis sp.*	*- E. coli*	*- Blastocystis sp.*<br>*- P. hominis*<br>*- E. hominis*<br>*- E. coli*	NEG	NEG	POS<br>(27.98)	NEG	NEG	NEG	-	-	-
DF058	V	*- Cysts*	*- E. nana*	*- E. nana*<br>*- S. mansonii (eggs)*	NEG	NEG	NEG	NEG	NEG	NEG	NEG	-	-	-
DF059	V	NEG	NEG	NEG	NEG	NEG	NEG	NEG	NEG	NEG	NEG	-	-	-
DF060	V	NEG	NEG	NEG	NEG	NEG	NEG	NEG	NEG	NEG	NEG	-	-	-
DF061	V	NEG	NEG	NEG	*- Blastocystis sp.*	NEG	NEG	POS<br>(32.03)	NEG	NEG	NEG	-	-	-
DF062	VI	NEG	NEG	NEG	NEG	NEG	NEG	NEG	NEG	NEG	NEG	-	-	-
DF063	II	NEG	NEG	NEG	NEG	NEG	NEG	NEG	NEG	NEG	NEG	-	-	-
DF064	II	NEG	NEG	NEG	*- Blastocystis sp.*	NEG	NEG	POS<br>(30.14)	NEG	NEG	NEG	-	-	-
DF065	II	*- Blastocystis sp.*	*- Blastocystis sp.*	NEG	*- Blastocystis sp.*	NEG	NEG	POS<br>(32.72)	NEG	NEG	NEG	-	-	-
DF066	II	*- Blastocystis sp.*	*- Blastocystis sp.*	NEG	NEG	NEG	NEG	POS<br>(31.78)	POS<br>(35.69)	NEG	NEG	-	-	-
DF067	II	*- E. coli*<br>*- E. histolyticia/dispar*<br>*- E. nana*	*- E. coli*<br>*- E. histolyticia/dispar*<br>*- E. nana*	*- E. coli*<br>*- E. histolyticia/dispar*<br>*- E. nana*	*- E. coli*<br>*- P. hominis*<br>*- Blastocystis sp.*	NEG	NEG	POS<br>(31.19)	NEG	NEG	NEG	POS	-	-
DF068	II	*- Blastocystis sp.*	*- D. fragilis*	NEG	*- Blastocystis sp.*	POS<br>(25.31)	NEG	POS<br>(30.45)	NEG	NEG	NEG	-	-	-
DF069	II	NEG	NEG	NEG	NEG	NEG	NEG	NEG	NEG	NEG	NEG	-	-	-
DF070	II	NEG	*- N. americanus*<br>*- A. lumbricoides*<br>*- Blastocystis sp.*	*- N. americanus*<br>*- A. lumbricoides*	*- A. lumbricoides (eggs)*	NEG	NEG	POS<br>(34.33)	NEG	NEG	NEG	-	NEG	POS
DF071	II	NEG	NEG	NEG	NEG	NEG	NEG	NEG	NEG	NEG	NEG	-	-	-
DF072	II	*- G. intestinalis*	*- G. intestinalis*	*- G. intestinalis*	*- G. intestinalis*	NEG	NEG	NEG	NEG	NEG	NEG	-	-	-
DF073	II	NEG	NEG	NEG	NEG	NEG	NEG	NEG	NEG	NEG	NEG	-	-	-
DF074	II	NEG	NEG	NEG	*- P. hominis*	NEG	NEG	NEG	NEG	NEG	NEG	-	-	-

DF075	II	NEG	*- Blastocystis sp.*	*- N. americanus (eggs)*	*- N. americanus (eggs)*	NEG	NEG	POS (37.16)	NEG	NEG	NEG	-	POS	NEG
DF076	IV	NEG	NEG	NEG	NEG	NEG	NEG	NEG	NEG	NEG	NEG	-	-	-
DF077	II	NEG	NEG	NEG	NEG	NEG	NEG	NEG	NEG	NEG	NEG	-	-	-
DF078	II	NEG	NEG	NEG	NEG	NEG	NEG	NEG	NEG	NEG	NEG	-	-	-
DF079	I	NEG	NEG	NEG	NEG	NEG	NEG	NEG	POS (43.38)	NEG	NEG	-	-	-
DF080	II	NEG	NEG	NEG	NEG	NEG	NEG	NEG	NEG	NEG	NEG	-	-	-
DF081	I	NEG	NEG	NEG	NEG	NEG	NEG	NEG	NEG	NEG	NEG	-	-	-
DF082	IV	*- Blastocystis sp.*	*- Blastocystis sp.*	NEG	NEG	NEG	NEG	POS (34.70)	NEG	NEG	NEG	-	-	-
DF083	VII	NEG	NEG	NEG	NEG	NEG	NEG	NEG	NEG	NEG	NEG	-	-	-
DF084	VII	NEG	*- Ancylostomidae (eggs)*	*- Ancylostomidae (eggs)*	*- Ancylostomidae (eggs)*	NEG	NEG	NEG	NEG	NEG	NEG	-	NEG	NEG
DF085	II	*- Blastocystis sp.*	*- Blastocystis sp.*	NEG	NEG	NEG	NEG	POS (31.54)	NEG	NEG	NEG	-	-	-
DF086	II	*- E. coli*	*- E. coli*	NEG	*- E. coli*<br>*- Blastocystis sp.*	NEG	NEG	POS (32.56)	NEG	NEG	NEG	-	-	-
DF087	VII	NEG	NEG	NEG	NEG	NEG	NEG	NEG	NEG	NEG	NEG	-	-	-
DF088	V	NEG	*- Blastocystis sp.*	NEG	*- Blastocystis sp.*	NEG	NEG	POS (> 40.00)	NEG	NEG	NEG	-	-	-
DF089	II	*- Blastocystis sp.*<br>*- Amoebas*<br>*- E. histolytica/dispar*	*- Blastocystis sp.*<br>*- E. hartmanni*<br>*- E. histolytica/dispar*	NEG	*- Blastocystis sp.*<br>*- E. hartmanni*<br>*- E. histolytica/dispar*	NEG	NEG	POS (31.34)	NEG	NEG	NEG	POS	-	-
DF090	I	NEG	*- Blastocystis sp.*	NEG	*- Blastocystis sp.*	NEG	NEG	POS (37.37)	NEG	NEG	NEG	-	-	-
DF091	V	NEG	NEG	NEG	NEG	NEG	NEG	NEG	NEG	NEG	NEG	-	-	-
DF092	V	*- Blastocystis sp.*<br>*- C. mesnili*<br>*- Amoebas*	*- Blastocystis sp.*<br>*- C. mesnili*<br>*- E. nana*	NEG	*- Blastocystis sp.*<br>*- C. mesnili*	NEG	NEG	POS (34.30)	NEG	NEG	POS (38.39)	-	-	-
DF093	V	NEG	NEG	NEG	*- Blastocystis sp.*	NEG	NEG	POS (> 40.00)	NEG	NEG	NEG	-	-	-
DF094	V	*- Blastocystis sp.*	*- Blastocystis sp.*	NEG	*- Blastocystis sp.*	NEG	NEG	POS (36.58)	NEG	NEG	NEG	-	-	-

DF095	V	*- Blastocystis sp.*	*- Blastocystis sp.*	NEG	*- P. hominis*<br>*- Blastocystis sp.*<br>*- E. intestinalis*<br>*- D. fragilis*<br>*- E. histolytica/dispar*	POS (30.87)	NEG	POS (30.45)	NEG	NEG	POS (35.77)	POS	-	-
DF096	V	*- D. fragilis*<br>*- E. hominis*<br>*- Blastocystis sp.*	*- D. fragilis*<br>*- E. hominis*<br>*- Blastocystis sp.*	NEG	*- E. intestinalis*<br>*- E. hominis*<br>*- Blastocystis sp.*	NEG	NEG	POS (36.21)	NEG	NEG	NEG	-	-	-
DF097	V	NEG	*- Blastocystis sp.*	NEG	*- Blastocystis sp.*	NEG	NEG	POS (33.83)	POS (35.78)	NEG	NEG	-	-	-
DF098	V	*- Blastocystis sp.*<br>*- E. histolytica/dispar*	*- Blastocystis sp.*<br>*- E. histolytica/dispar*<br>*- E. hartmanni*	*- E. histolytica/dispar*	*- Blastocystis sp.*<br>*- E. histolytica/dispar*<br>*- Amoebas*	NEG	NEG	POS (27.11)	NEG	NEG	NEG	POS	-	-
DF099	V	NEG	NEG	NEG	NEG	NEG	NEG	NEG	NEG	NEG	NEG	-	-	-
DF100	V	NEG	NEG	NEG	NEG	NEG	NEG	NEG	NEG	NEG	NEG	-	-	-
DF101	V	NEG	NEG	NEG	NEG	NEG	NEG	NEG	NEG	NEG	NEG	-	-	-
DF102	V	*- G. intestinalis*<br>*- Blastocystis sp.*	*- G. intestinalis*<br>*- Blastocystis sp.*	*- G. intestinalis*	*- G. intestinalis*	NEG	NEG	POS (32.42)	NEG	NEG	NEG	-	-	-
DF103	V	NEG	NEG	NEG	NEG	NEG	NEG	NEG	NEG	NEG	NEG	-	-	-
DF104	V	*- Amoebas*<br>*- E. coli*	*- Blastocystis sp.*<br>*- E. nana*<br>*- D. fragilis*	*- E. nana*	*- Blastocystis sp.*	NEG	NEG	POS (28.23)	NEG	NEG	NEG	-	-	-
DF105	V	*- Cysts*	NEG	NEG	*- Blastocystis sp.*	NEG	NEG	POS (34.49)	NEG	NEG	NEG	-	-	-
DF106	V	NEG	*- Blastocystis sp.*	NEG	NEG	NEG	NEG	POS (29.47)	NEG	NEG	NEG	-	-	-
DF107	V	NEG	*- Blastocystis sp.*	NEG	*- Blastocystis sp.*	NEG	NEG	POS (32.73)	NEG	NEG	NEG	-	-	-
DF108	VII	NEG	*- Blastocystis sp.*	NEG	NEG	NEG	NEG	POS (29.49)	NEG	NEG	NEG	-	-	-
DF109	IV	NEG	NEG	NEG	NEG	NEG	NEG	NEG	NEG	NEG	NEG	-	-	-
DF110	V	NEG	NEG	NEG	NEG	NEG	NEG	NEG	NEG	NEG	NEG	-	-	-

DF111	V	*- Blastocystis sp.*<br>*- Amoeba*<br>*- D. fragilis*	*- E. histolytica/dispar*<br>*- G. intestinalis*<br>*- E. nana*	*- E. coli*<br>*- E. histolytica/dispar*	*- E. histolytica/dispar*<br>*- E. coli*<br>*- Blastocystis sp.*	POS<br>(30.54)	NEG	POS<br>(32.87)	NEG	NEG	NEG	POS	-	-
DF112	V	NEG	*- E. coli*<br>*- Blastocystis sp.*	NEG	*- E. intestinalis*<br>*- E. coli*<br>*- Blastocystis sp.*	NEG	NEG	POS<br>(35.85)	NEG	NEG	NEG	-	-	-
DF113	V	*- E. coli*	*- E. coli*	*- E. coli*	*- E. coli*	POS<br>(21.98)	NEG	NEG	NEG	NEG	NEG	-	-	-
DF114	V	*- D. fragilis*	*- D. fragilis*<br>*- E. nana*<br>*- E. histolytica/dispar*<br>*- Blastocystis sp.*	NEG	*- D. fragilis*<br>*- Amoeba*	POS<br>(28.78)	NEG	POS<br>(29.46)	NEG	NEG	NEG	POS	-	-
DF115	V	*- G. intestinalis*<br>*- Blastocystis sp.*	*- G. intestinalis*<br>*- Blastocystis sp.*	*- G. intestinalis*	*- G. intestinalis*<br>*- Blastocystis sp.*<br>*- D. fragilis*	NEG	NEG	POS<br>(29.42)	NEG	NEG	NEG	-	-	-
DF116	V	*- G. intestinalis*	*- G. intestinalis*	*- G. intestinalis*	*- G. intestinalis*	NEG	NEG	NEG	NEG	NEG	NEG	-	-	-
DF117	V	*- H. nana (eggs)*	*- H. nana (eggs)*<br>*- E. hartmanni*<br>*- Blastocystis sp.*<br>*- G. intestinalis*<br>*- E. coli*<br>*-E. nana*	*- H. nana (eggs)*	*- H. nana (eggs)*<br>*- E. hartmanni*<br>*- Blastocystis sp.*<br>*- G. intestinalis*<br>*- E. coli*<br>*-E. nana*	NEG	NEG	POS<br>(32.56)	NEG	NEG	NEG	-	-	-
DF118	VII	NEG	NEG	NEG	NEG	NEG	NEG	NEG	NEG	NEG	NEG	-	-	-
DF119	V	NEG	*- Blastocystis sp.*<br>*- E. hartmanni*<br>*- E. histolytica/dispar*	*- E. histolytica/dispar*	*- Blastocystis sp.*	NEG	NEG	POS<br>(> 40.00)	NEG	NEG	NEG	POS	-	-
DF120	VII	NEG	NEG	NEG	NEG	NEG	NEG	NEG	NEG	NEG	NEG	-	-	-
DF121	VI	NEG	NEG	NEG	NEG	NEG	NEG	NEG	NEG	NEG	NEG	-	-	-
DF122	VI	*- E. nana*<br>*- E. coli*<br>*- E. hartmanni*<br>*- Blastocystis sp.*	*- Blastocystis sp.*<br>*- E. coli*<br>*- E. histolytica/dipar*<br>*- G. intestinalis*	*- Cysts*<br>*- E. coli*	*- G. intestinalis*<br>*- Amoeba*	NEG	NEG	POS<br>(33.85)	NEG	NEG	NEG	POS	-	-
DF123	VI	*- Blastocystis sp.*	*- Blastocystis sp.*	NEG	NEG	NEG	NEG	POS<br>(21.23)	NEG	NEG	NEG	-	-	-

DF124	VI	*- G. intestinalis*<br>*- E. coli*<br>*- Amoeba*	*- G. intestinalis*<br>*- E. hartmanni*	*- G. intestinalis*	*- E. coli*<br>*- G. intestinalis*<br>*- Blastocystis sp.*<br>*- E. hominis*<br>*- D. fragilis*<br>*- Amoeba*	POS<br>(29.20)	NEG	POS<br>(35.25)	NEG	NEG	NEG	-	-	-
DF125	VI	NEG	NEG	NEG	*- E. histolytica/dipar*<br>*- Blastocystis sp.*	NEG	NEG	POS<br>(39.91)	NEG	NEG	NEG	POS	-	-
DF126	VI	NEG	NEG	NEG	NEG	NEG	NEG	NEG	NEG	NEG	NEG	-	-	-
DF127	VI	*- E. coli*	*- E. coli*<br>*- E. nana*<br>*- Blastocystis sp.*	*- E. coli*	*- E. coli*<br>*- Blastocystis sp.*	NEG	NEG	POS<br>(25.54)	NEG	NEG	NEG	-	-	-
DF128	VI	*- E. histolytica/dispar*	NEG	NEG	NEG	NEG	NEG	NEG	NEG	NEG	NEG	-	-	-
DF129	V	*- G. intestinalis*	*- G. intestinalis*<br>*- Blastocystis sp.*	*- G. intestinalis*	*- G. intestinalis*<br>*- Blastocystis sp.*	NEG	NEG	POS<br>(32.31)	NEG	NEG	NEG	-	-	-
DF130	II	NEG	NEG	NEG	NEG	NEG	NEG	NEG	NEG	NEG	NEG	-	-	-
DF131	V	NEG	NEG	NEG	NEG	NEG	NEG	NEG	NEG	NEG	NEG	-	-	-
DF132	V	NEG	NEG	NEG	NEG	NEG	NEG	NEG	NEG	NEG	NEG	-	-	-
DF133	V	*- E. coli*	*- E. coli*	NEG	*- E. coli*	NEG	NEG	NEG	NEG	NEG	NEG	-	-	-
DF134	V	NEG	NEG	NEG	NEG	NEG	NEG	NEG	NEG	NEG	NEG	-	-	-
DF135	V	*- E. histolytica/dispar*<br>*- D. fragilis*	*- E. coli*<br>*- E. hartmanni*	NEG	*- E. histolytica/dispar*	NEG	NEG	NEG	NEG	NEG	POS<br>(35.96)	POS	-	-
DF136	V	*- D. fragilis*	*- D. fragilis*	*- H. nana (eggs)*	*- D. fragilis*<br>*- H. nana (eggs)*	POS<br>(24.84)	NEG	NEG	NEG	NEG	NEG	-	-	-
DF137	V	*- E. coli*<br>*- C. mesnili*<br>*- G. intestinalis*	*- E. coli*<br>*- G. intestinalis*<br>*- Blastocystis sp.*	*- E. coli*<br>*- G. intestinalis*	*- E. coli*<br>*- G. intestinalis*<br>*- Blastocystis sp.*	NEG	NEG	POS<br>(31.32)	NEG	NEG	NEG	-	-	-
DF138	V	NEG	*- Blastocystis sp.*	NEG	*- Blastocystis sp.*	NEG	NEG	POS<br>(25.45)	NEG	NEG	NEG	-	-	-

DF139	V	*- Blastocystis sp.*<br>*- E. hominis*	*- Blastocystis sp.*	NEG	*- Blastocystis sp.*<br>*- E. hominis*	NEG	NEG	POS<br>(> 40.00)	NEG	POS<br>(35.93)	POS<br>(35.51)	-	-	-
DF140	V	*- Blastocystis sp.*<br>*- D. fragilis*<br>*- Amoebas*	*- Blastocystis sp.*<br>*- E. hartmanni*<br>*- Amoebas*	*- Blastocystis sp.*<br>*- D. fragilis*	*- E. coli*<br>*- Blastocystis sp.*	POS<br>(30.21)	NEG	POS<br>(32.95)	NEG	NEG	NEG	-	-	-
DF141	V	NEG	*- Blastocystis sp.*	NEG	*- Blastocystis sp.*	NEG	NEG	POS<br>(> 40.00)	POS<br>(42.55)	NEG	NEG	-	-	-
DF142	V	NEG	NEG	NEG	NEG	NEG	NEG	NEG	NEG	NEG	NEG	-	-	-
DF143	I	NEG	NEG	NEG	NEG	NEG	NEG	NEG	NEG	NEG	NEG	-	-	-
DF144	II	*- Blastocystis sp.*	*- Blastocystis sp.*	NEG	*- Blastocystis sp.*	NEG	NEG	POS<br>(30.31)	NEG	NEG	NEG	-	-	-
DF145	VII	NEG	NEG	NEG	NEG	NEG	NEG	NEG	NEG	NEG	NEG	-	-	-
DF146	V	*- E. histolytica/dispar*	*- E. histolytica/dispar*<br>*- Blastocystis sp.*	NEG	NEG	NEG	NEG	POS<br>(26.22)	NEG	NEG	NEG	POS	-	-
DF147	III	NEG	NEG	NEG	NEG	NEG	NEG	NEG	NEG	NEG	NEG	-	-	-
DF148	III	*- E. coli*<br>*- Cysts*	*- E. coli*<br>*- E. nana*<br>*- C. mesnili*	*- E. coli*<br>*- Cysts*	*- E. coli*<br>*- E. intestinalis*<br>*- C. mesnili*<br>*- Amoebas*<br>*- Cysts*	NEG	NEG	NEG	NEG	NEG	NEG	-	-	-
DF149	III	*- Blastocystis sp.*	*- Blastocystis sp.*	NEG	NEG	NEG	NEG	POS<br>(31.30)	NEG	NEG	NEG	-	-	-
DF150	III	NEG	NEG	NEG	NEG	NEG	NEG	POS<br>(34.61)	NEG	NEG	NEG	-	-	-
DF151	III	*- E. coli*<br>*- Cysts*	*- E. coli*<br>*- E. nana*<br>*- Blastocystis sp.*	*- E. coli*	*- E. coli*<br>*- Blastocystis sp.*	NEG	NEG	POS<br>(23.07)	NEG	NEG	NEG	-	-	-
DF152	III	NEG	NEG	NEG	NEG	NEG	NEG	POS<br>(29.75)	NEG	NEG	NEG	-	-	-
DF153	III	*- C. mesnili*<br>*- E. histolytica/dispar*<br>*- E. hominis*<br>*- Blastocystis sp.*	*- C. mesnili*<br>*- E. histolytica/dispar*<br>*- E. hominis*<br>*- E. hartmanni*<br>*- Blastocystis sp.*	NEG	*- Blastocystis sp.*<br>*- D. fragilis*<br>*- Amoebas*	NEG	NEG	POS<br>(32.57)	NEG	NEG	NEG	POS	-	-

DF154	III	*- Blastocystis sp.*<br>*- E. coli*<br>*- D . fragilis*	*- Blastocystis sp.*<br>*- E. hartmanni*	NEG	*- Blastocystis sp.*	NEG	NEG	POS<br>(34.89)	NEG	NEG	NEG	-	-	-
DF155	III	*- E. coli*	*- Blastocystis sp.*<br>*- E. coli*<br>*- E. hartmanii*	NEG	*- Blastocystis sp.*	NEG	NEG	POS<br>(32.73)	NEG	NEG	NEG	-	-	-
DF156	III	*- Blastocystis sp.*	*- Blastocystis sp.*	NEG	*- Blastocystis sp.*	NEG	NEG	POS<br>(34.62)	NEG	NEG	NEG	-	-	-
DF157	III	*- Blastocystis sp.*	*- Blastocystis sp.*	*- Blastocystis sp.*	NEG	NEG	NEG	POS<br>(31.56)	NEG	NEG	NEG	-	-	-
DF158	III	NEG	*- Blastocystis sp.*	NEG	*- Blastocystis sp.*	NEG	NEG	POS<br>(29.26)	NEG	NEG	NEG	-	-	-
DF159	III	*- C. mesnili*<br>*- E. hominis*	*- C. mesnili*<br>*- Blastocystis sp.*	NEG	*- Blastocystis sp.*<br>*- P. hominis*<br>*- E. hominis*	POS<br>(24.10)	NEG	POS<br>(32.96)	NEG	NEG	NEG	-	-	-
DF160	I	NEG	NEG	NEG	NEG	NEG	NEG	POS<br>(34.19)	NEG	NEG	NEG	-	-	-
DF161	IV	*- E. coli*	*- E. nana*	*- T. trichiura*<br>*- E. coli*	*- Blastocystis sp.*<br>*- E. coli*	NEG	NEG	POS<br>(28.25)	NEG	NEG	POS<br>(41.44)	-	-	-
DF162	IV	*- C. mesnili*<br>*- Blastocystis sp.*<br>*- Ancylostomidae (eggs)*	*- C. mesnili*<br>*- Blastocystis sp.*<br>*- Ancylostomidae (eggs)*<br>*- E. histolytica/dispar*<br>*- E. hartmanni*	*- Blastocystis sp.*<br>*- Ancylostomidae (eggs)*	*- Blastocystis sp.*<br>*- E. coli*	NEG	NEG	POS<br>(33.66)	NEG	NEG	NEG	POS	NEG	NEG
DF163	IV	*- G. intestinalis*<br>*- C. mesnili*<br>*- Blastocystis sp.*	*- G. intestinalis*<br>*- Blastocystis sp.*	*- G. intestinalis*<br>*- Nematod (larvae)*	*- G. intestinalis*<br>*- C. mesnili*<br>*- Blastocystis sp.*	NEG	NEG	POS<br>(28.47)	NEG	NEG	NEG	-	-	-
DF164	IV	NEG	NEG	NEG	NEG	NEG	NEG	NEG	NEG	NEG	NEG	-	-	-
DF165	IV	*- G. intestinalis*<br>*- E. coli*	*- G. intestinalis*<br>*- E. coli*	*- G. intestinalis*<br>*- E. coli*	*- Blastocystis sp.*<br>*- E. hominis*<br>*- E. coli*	POS<br>(30.27)	NEG	POS<br>(29.34)	NEG	NEG	NEG	-	-	-

DF167	III	*- D. fragilis ?*<br>*- E. coli*<br>*- B. coli*<br>*- Blastocystis sp. ?*<br>*- C. mesnili*<br>*- Cysts*	*- Blastocystis sp.*<br>*- C. mesnili*<br>*- E. coli*<br>*- E. nana*<br>*- E. hartmanii*	*- E. coli*	*- Blastocystis sp. ?*<br>*- C. mesnili*<br>*- E. coli*<br>*- Flagellate*	POS<br>(30.13)	NEG	POS<br>(29.26)	NEG	POS<br>(36.69)	NEG	-	-	-
DF168	III	NEG	*- E. coli*<br>*- Blastocystis sp.*<br>*- C. mesnili*	*- C. mesnili*<br>*- E. coli*<br>*- T. trichiura (eggs)*	*- C. mesnili*<br>*- E. coli*<br>*- T. trichiura (eggs)*<br>*- E. intestinalis*<br>*- E. hominis*	NEG	NEG	POS<br>(32.17)	NEG	NEG	NEG	-	-	-
DF169	III	NEG	NEG	*- E. coli*	NEG	NEG	NEG	NEG	NEG	NEG	NEG	-	-	-
DF170	III	NEG	NEG	*- T. trichiura*	NEG	NEG	NEG	NEG	NEG	NEG	NEG	-	-	-
DF171	III	*- E. coli*<br>*- C. mesnili*	*- E. coli*<br>*- Blastocystis sp.*<br>*- C. mesnili*	*- E. coli*<br>*- C. mesnili*	*- E. intestinalis*<br>*- Blastocystis sp.*<br>*- C. mesnili*<br>*- P. hominis*<br>*- E. histolyticia/dispar*	NEG	POS<br>(28.68)	POS<br>(35.22)	NEG	NEG	NEG	POS	-	-
DF172	III	NEG	NEG	NEG	*- Blastocystis sp.*	NEG	NEG	POS<br>(32.25)	NEG	NEG	NEG	-	-	-
DF173	III	NEG	NEG	NEG	NEG	NEG	NEG	POS<br>(30.23)	NEG	NEG	NEG	-	-	-
DF174	III	NEG	*- Blastocystis sp. ?*	NEG	NEG	NEG	NEG	POS<br>(31.96)	NEG	POS<br>(36.44)	NEG	-	-	-
DF175	III	NEG	NEG	*- E. coli*	NEG	POS<br>(30.90)	NEG	POS<br>(27.69)	NEG	NEG	NEG	-	-	-
DF176	IV	*- E. coli*<br>*- E. histolytica/dispar*<br>*- Cysts*	*- E. nana*<br>*- C. mesnili*<br>*- Blastocystis sp.*<br>*- E. coli*	*- E. nana*<br>*- C. mesnili*<br>*- Blastocystis sp.*<br>*- E. coli*	*- E. histolytica/dispar*<br>*- C. mesnili*<br>*- Blastocystis sp.*<br>*- E. coli*	NEG	NEG	POS<br>(32.77)	NEG	NEG	NEG	POS	-	-
DF177	IV	*- C. mesnili*	*- C. mesnili*	*- C. mesnili*	*- C. mesnili*<br>*- Blastocystis sp.*	NEG	NEG	POS<br>(30.82)	NEG	NEG	NEG	-	-	-
DF178	IV	*- E. coli*<br>*- C. mesnili*	*- E. coli*<br>*- C. mesnili*<br>*- E. histolytica/dispar*	*- E. coli*	*- E. coli*<br>*- C. mesnili*<br>*- E. histolytica/dispar*	NEG	NEG	POS<br>(> 40.00)	NEG	NEG	NEG	POS	-	-
DF179	IV	*- Blastocystis sp.*	*- Blastocystis sp.*	*- E. coli*	*- Blastocystis sp.*	NEG	NEG	POS<br>(32.67)	NEG	NEG	NEG	-	-	-

DF180	IV	*- Blastocystis sp.*<br>*- G. intestinalis*<br>*- C. mesnili*<br>*- Amoebas*	*- Blastocystis sp.*<br>*- G. intestinalis*<br>*- C. mesnili*<br>*- E. nana*	*- G. intestinalis*<br>*- C. mesnili*<br>*- E. coli*	*- Blastocystis sp.*<br>*- G. intestinalis*<br>*- C. mesnili*	NEG	NEG	POS<br>(28.17)	NEG	NEG	NEG	-	-	-
DF181	III	*- Blastocystis sp.*<br>*- D. fragilis*	*- Blastocystis sp.*<br>*- D. fragilis*<br>*- E. nana*	*- C. mesnili*<br>*- E. hartmanni*	*- Blastocystis sp.*<br>*- D. fragilis*<br>*- E. nana*<br>*- Amoebas*	NEG	NEG	POS<br>(32.77)	NEG	NEG	NEG	-	-	-
DF182	III	*- Blastocystis sp.*<br>*- C. mesnili*	*- Blastocystis sp.*	*- C. mesnili*	*- Blastocystis sp.*	NEG	NEG	POS<br>(37.07)	NEG	NEG	NEG	-	-	-
DF183	III	*- Blastocystis sp.*<br>*- E. coli*<br>*- Amoebas*	*- Blastocystis sp.*<br>*- E. coli*	*- Cysts*<br>*- E. coli*	*- Blastocystis sp.*<br>*- E. coli*	NEG	NEG	POS<br>(33.99)	NEG	NEG	NEG	-	-	-
DF184	III	*- E. coli*	*- E. coli*	*- E. coli*	*- Blastocystis sp.*<br>*- E. coli*<br>*- Amoebas*	NEG	NEG	POS<br>(21.50)	NEG	NEG	NEG	-	-	-
DF185	III	*- Blastocystis sp.*	*- Blastocystis sp.*	NEG	*- Blastocystis sp.*	NEG	NEG	POS<br>(29.62)	NEG	NEG	NEG	-	-	-
DF186	IV	*- Cysts*	*- E. coli*	*- E. coli*	*- Cysts*	POS<br>(24.42)	NEG	POS<br>(26.86)	NEG	NEG	POS<br>(29.81)	-	-	-
DF187	III	*- Blastocystis sp.*	*- Blastocystis sp.*	NEG	NEG	NEG	NEG	POS<br>(27.13)	NEG	NEG	NEG	-	-	-
DF188	IV	*- Blastocystis sp.*	*- Blastocystis sp.*<br>*- H. nana*	*- H. nana*	*- H. nana*	NEG	NEG	POS<br>(28.63)	NEG	NEG	NEG	-	-	-
DF189	IV	*- Cysts*	NEG	NEG	NEG	POS<br>(28.06)	NEG	POS<br>(31.27)	NEG	NEG	NEG	-	-	-
DF190	IV	*- Blastocystis sp.*<br>*- G. intestinalis*<br>*- Cysts*	*- Blastocystis sp.*<br>*- G. intestinalis*	*- H. nana*<br>*- E. coli*	*- Blastocystis sp.*<br>*- G. intestinalis*	POS<br>(25.89)	NEG	POS<br>(22.57)	NEG	NEG	NEG	-	-	-
DF191	IV	*- Blastocystis sp.*<br>*- C. mesnili*<br>*- D. fragilis*<br>*- Amoebas*	*- Blastocystis sp.*<br>*- E. histolytica/dispar*<br>*- D. fragilis*	*- Ancylostomidae (eggs)*	*- Blastocystis sp.*<br>*- C. mesnili*<br>*- E. hominis*<br>*- E. coli*<br>*- D. fragilis*<br>*- E. histolytica/dispar*	NEG	NEG	POS<br>(28.56)	NEG	NEG	NEG	POS	NEG	NEG

DF192	IV	*- Blastocystis sp.*	NEG	NEG	*- Blastocystis sp.*<br>*- E. hominis*	NEG	NEG	POS<br>(29.28)	NEG	NEG	NEG	-	-	-
DF193	I	NEG	*- Blastocystis sp.*	NEG	NEG	NEG	NEG	POS<br>(39.95)	NEG	NEG	NEG	-	-	-
DF194	VII	NEG	NEG	NEG	NEG	NEG	NEG	NEG	NEG	NEG	NEG	-	-	-
DF195	IV	*- Blastocystis sp.*<br>*- Cysts*	*- Blastocystis sp.*	*- Cysts*	*- Blastocystis sp.*	NEG	NEG	POS<br>(24.62)	NEG	NEG	NEG	-	-	-
DF196	IV	*- E. coli*<br>*- E. histolytica/dispar*<br>*- D. fragilis ?*	*- Blastocystis sp.*<br>*- C. mesnili*<br>*- E. nana*<br>*- E. histolytica/dispar*	*- E. coli*<br>*- Cysts*	*- Blastocystis sp.*<br>*- C. mesnili*<br>*- E. coli*<br>*- E. histolytica/dispar*<br>*- Cysts*	POS<br>(29.84)	NEG	POS<br>(26.31)	NEG	NEG	NEG	POS	-	-
DF197	IV	*- Blastocystis sp.*	*- Blastocystis sp.*	*- Blastocystis sp.*	*- Blastocystis sp.*	NEG	NEG	POS<br>(36.11)	NEG	NEG	NEG	-	-	-
DF198	IV	NEG	*- Blastocystis sp.*	NEG	*- Blastocystis sp.*<br>*- C. mesnili*<br>*- E. hominis*	NEG	NEG	POS<br>(30.10)	NEG	NEG	NEG	-	-	-
DF199	IV	NEG	NEG	NEG	NEG	NEG	NEG	POS<br>(33.90)	NEG	NEG	NEG	-	-	-
DF200	IV	NEG	NEG	NEG	NEG	NEG	NEG	POS<br>(25.12)	NEG	NEG	NEG	-	-	-
DF201	IV	*- Blastocystis sp.*<br>*- E. coli*<br>*- Cysts*	*- Blastocystis sp.*<br>*- E. coli*<br>*- G. intestinalis*	*- Blastocystis sp.*<br>*- E. coli*<br>*- G. intestinalis*	*- Blastocystis sp.*<br>*- E. coli*<br>*- G. intestinalis*<br>*- C. mesnili*<br>*- D. fragilis*	POS<br>(29.59)	NEG	NEG	NEG	NEG	NEG	-	-	-
DF202	IV	*- Blastocystis sp.*<br>*- G. intestinalis*	NEG	*- Blastocystis sp.*	NEG	NEG	NEG	POS<br>(27.26)	NEG	NEG	NEG	-	-	-
DF203	IV	NEG	NEG	*- Ancylostomidae (eggs)*	NEG	NEG	NEG	POS<br>(30.45)	NEG	NEG	NEG	-	NEG	NEG
DF204	IV	*- G. intestinalis*	*- G. intestinalis*	NEG	*- G. intestinalis*	NEG	NEG	POS<br>(25.56)	NEG	NEG	NEG	-	-	-
DF205	IV	*- Cysts*	*- Blastocystis sp.*<br>*- C. mesnili*	*- Cysts*	*- Blastocystis sp.*<br>*- E. histolytica/dispar*	NEG	NEG	POS<br>(29.78)	NEG	NEG	NEG	POS	-	-
DF206	III	*- Blastocystis sp.*	*- Blastocystis sp.*<br>*- E. coli*	NEG	*- Blastocystis sp.*	NEG	NEG	POS<br>(25.33)	NEG	NEG	NEG	-	-	-

DF207	III	- *Cysts*	- *Ancylostomidae (eggs)*	- *Ancylostomidae (eggs)*<br>- *E. coli*	- *Blastocystis sp.*<br>- *E. hominis*	NEG	NEG	POS<br>(28.43)	NEG	NEG	NEG	-	POS	NEG
DF208	III	- *Blastocystis sp.*	- *Blastocystis sp.*	NEG	- *Blastocystis sp.*	NEG	NEG	POS<br>(25.64)	NEG	NEG	NEG	-	-	-
DF209	III	- *Blastocystis sp.*	- *Blastocystis sp.*<br>- *D. fragilis*<br>- *E. nana*	- *Blastocystis sp.*	- *Blastocystis sp.*	NEG	NEG	POS<br>(23.63)	NEG	NEG	NEG	-	-	-
DF210	III	- *E. coli*	- *Blastocystis sp.*<br>- *E. coli*	- *E. coli*	- *Blastocystis sp.*<br>- *E. coli*	NEG	NEG	POS<br>(26.49)	NEG	POS<br>(40.69)	NEG	-	-	-
DF211	III	- *Blastocystis sp.*	- *Blastocystis sp.*	NEG	NEG	NEG	NEG	POS<br>(28.90)	NEG	NEG	NEG	-	-	-
DF212	III	NEG	- *Blastocystis sp.*	NEG	- *Blastocystis sp.*	NEG	NEG	POS<br>(23.30)	NEG	NEG	NEG	-	-	-
DF213	I	NEG	- *Blastocystis sp.*	- *H. nana (eggs)*	NEG	POS<br>(26.11)	POS<br>(29.31)	POS<br>(26.46)	NEG	NEG	NEG	-	-	-
DF214	I	- *Blastocystis sp.*	- *Blastocystis sp.*	NEG	NEG	POS<br>(29.35)	NEG	POS<br>(27.77)	NEG	NEG	NEG	-	-	-
DF215	I	- *E. histolytica/dispar*<br>- *E. coli*	- *Blastocystis sp.*	NEG	- *Blastocystis sp.*<br>- *E. histolytica/dispar*<br>- *E. coli*	NEG	NEG	POS<br>(18.43)	NEG	NEG	NEG	POS	-	-
DF216	I	NEG	NEG	NEG	- *E. coli*	POS<br>(29.48)	NEG	POS<br>(34.80)	NEG	NEG	NEG	-	-	-
DF217	I	NEG	NEG	NEG	NEG	NEG	NEG	NEG	NEG	NEG	NEG	-	-	-
DF218	V	NEG	- *E. nana*<br>- *G. intestinalis*	NEG	NEG	NEG	NEG	NEG	NEG	NEG	NEG	-	-	-
DF219	III	NEG	NEG	- *E. coli*	NEG	NEG	NEG	POS<br>(26.84)	NEG	NEG	NEG	-	-	-
DF220	III	- *Blastocystis sp.*	NEG	- *E. coli*	- *Blastocystis sp.*	POS<br>(25.69)	NEG	POS<br>(20.77)	NEG	NEG	NEG	-	-	-
DF221	III	- *Blastocystis sp.*<br>- *E. coli*	NEG	- *Cysts*	- *Blastocystis sp.*<br>- *E. coli*<br>- *Cysts*	NEG	NEG	POS<br>(28.15)	NEG	NEG	NEG	-	-	-
DF222	III	- *E. coli*	- *Blastocystis sp.*<br>- *E. coli*	- *E. coli*	- *Blastocystis sp.*<br>- *E. coli*<br>- *E. hominis*<br>- *P. hominis*	NEG	NEG	POS<br>(35.72)	NEG	NEG	NEG	-	-	-

DF223	III	NEG	- *Blastocystis sp.*	NEG	- *Blastocystis sp.*	NEG	NEG	POS (30.55)	NEG	NEG	NEG	-	-	-
DF224	II	NEG	NEG	NEG	NEG	POS (35.38) (*S. similis*)	-	POS (28.74)	NEG	NEG	NEG	-	-	-
DF225	IV	- *E. coli* - *G. intestinalis*	- *E. coli* - *G. intestinalis*	- *E. coli*	- *E. coli* - *G. intestinalis*	POS (28.89)	NEG	POS (23.26	NEG	NEG	NEG	-	-	-
DF226	IV	NEG	NEG	NEG	NEG	NEG	NEG	POS (> 40.00)	NEG	NEG	NEG	-	-	-
DF227	IV	NEG	- *Blastocystis sp.*	NEG	NEG	NEG	NEG	POS (28.67)	NEG	NEG	NEG	-	-	-
DF228	IV	- *Blastocystis sp.* - *E. coli*	- *C. mesnili* - *E. coli*	- *E. coli*	- *Blastocystis sp.* - *E. coli* - *E. intestinalis* - *E. hominis*	POS (6.09)	NEG	POS (21.50)	NEG	NEG	NEG	-	-	-
DF229	IV	- *Ancylostomidae (eggs)*	NEG	- *Ancylostomidae (eggs)*	NEG	NEG	NEG	NEG	NEG	NEG	NEG	-	POS	NEG
DF230	IV	- *E. coli* - *G. intestinalis*	- *E. coli*	- *E. coli*	NEG	NEG	NEG	POS (31.80)	NEG	NEG	NEG	-	-	-
DF231	IV	- *Blastocystis sp.* - *E. hominis* - *E. hartmanni*	- *Blastocystis sp.* - *E. coli*	- *E. coli*	- *Blastocystis sp.* - *E. coli*	NEG	NEG	POS (23.32)	NEG	NEG	NEG	-	-	-
DF232	III	- *E. coli*	- *Blastocystis sp.* - *E. coli*	- *E. coli*	- *Blastocystis sp.* - *E. coli*	NEG	NEG	POS (20.11)	POS (36.18)	NEG	NEG	-	-	-
DF233	III	NEG	NEG	NEG	NEG	NEG	NEG	NEG	NEG	NEG	NEG	-	-	-
DF234	III	NEG	NEG	NEG	NEG	NEG	NEG	NEG	NEG	NEG	NEG	-	-	-
DF235	III	- *Blastocystis sp.* - *E. coli*	- *Blastocystis sp.* - *E. coli* - *E. nana*	- *E. coli*	- *Blastocystis sp.* - *E. coli*	NEG	NEG	POS (23.11)	NEG	NEG	NEG	-	-	-
DF236	III	- *Blastocystis sp.*	NEG	NEG	NEG	NEG	NEG	POS (32.49)	NEG	NEG	NEG	-	-	-
DF237	III	- *Blastocystis sp.*	- *Blastocystis sp.* - *Cysts*	- *E. coli*	NEG	NEG	POS (29.12)	POS (28.56)	NEG	NEG	NEG	-	-	-

DF238	III	*- Blastocystis sp.*<br>*- E. hominis*<br>*- E. coli*	*- Blastocystis sp.*<br>*- E. hominis*<br>*- E. coli*<br>*- E. nana*	*- E. coli*	*- Blastocystis sp.*<br>*- E. coli*<br>*- E. hominis*<br>*- C. mesnili*	NEG	NEG	POS<br>(26.97)	NEG	NEG	NEG	-	-	-
DF239	III	NEG	*- Blastocystis sp.*	NEG	*- Blastocystis sp.*	NEG	NEG	POS<br>(22.95)	NEG	NEG	NEG	-	-	-
DF240	III	*- Cysts*	*- E. intestinalis*<br>*- E. coli*<br>*- E. nana*<br>*- C. mesnili*	NEG	*- Blastocystis sp.*<br>*- E. coli*	NEG	NEG	POS<br>(22.94)	NEG	NEG	NEG	-	-	-
DF241	III	*- E. coli*<br>*- Blastocystis sp.*	*- E. coli*	*- E. coli*	*- E. coli*	NEG	NEG	POS<br>(25.80)	NEG	NEG	NEG	-	-	-
DF242	III	*- E. histolytica/dispar*	*- Blastocystis sp.*<br>*- E. hartmanni*	NEG	NEG	NEG	NEG	POS(	NEG	NEG	NEG	POS	-	-
DF243	III	NEG	*- Blastocystis sp.*	NEG	*- Blastocystis sp.*	NEG	NEG	POS<br>(27.96)	NEG	NEG	NEG	-	-	-
DF244	IV	*- C. mesnili*<br>*- Amoebas*<br>*- Flagellate*	NEG	NEG	*- Blastocystis sp.*<br>*- E. hominis*	NEG	NEG	POS<br>(27.52)	NEG	NEG	NEG	-	-	-
DF245	IV	NEG	*- Blastocystis sp.*<br>*- E. nana*	*- E. coli*	NEG	POS<br>(30.49)	NEG	POS<br>(25.87)	NEG	NEG	NEG	-	-	-
DF246	II	*- Blastocystis sp.*<br>*- E. hartmanni*<br>*- E. histolytica/dispar*<br>*- Flagellates*	*- Blastocystis sp.*<br>*- E. coli*<br>*- E. histolytica/dispar*	*- E. coli*<br>*- Cysts*	*- Blastocystis sp.*<br>*- E. coli*<br>*- C. mesnili*<br>*- E. hominis*<br>*- Amoebas*	POS<br>(25.66)	NEG	POS<br>(23.85)	NEG	NEG	NEG	POS	-	-
DF247	IV	NEG	*- Blastocystis sp.*	*- Cysts*	*- Blastocystis sp.*	NEG	NEG	POS<br>(29.20)	NEG	NEG	NEG	-	-	-
DF248	IV	*- E. coli*	NEG	*- E. coli*	*- E. coli*<br>*- Blastocystis sp.*	POS<br>(27.14)	NEG	POS<br>(26.87)	NEG	NEG	NEG	-	-	-
DF249	IV	*- I. butschlii*<br>*- E. coli*<br>*- Cysts*	*- I. butschlii*<br>*- E. coli*<br>*- E. histolytica/dispar*	*- I. butschlii*<br>*- E. coli*<br>*- Cysts*	*- I. butschlii*<br>*- E. coli*<br>*- Cysts*	POS<br>(29.72)	NEG	POS<br>(30.54)	NEG	NEG	NEG	POS	-	-
DF250	I	NEG	*- Blastocystis sp.*	*- T. trichiura (eggs)*	NEG	POS<br>(31.99)	NEG	POS<br>(20.89)	NEG	NEG	NEG	-	-	-

DF251	V	*- Blastocystis sp.*<br>*- Cysts*	*- Blastocystis sp.*	*- E. coli*<br>*- Cysts*	*- Blastocystis sp.*<br>*- C. mesnili*<br>*- E. hominis*	POS<br>(5.90)	NEG	POS<br>(34.16)	NEG	NEG	NEG	-	-	-
DF252	V	*- Blastocystis sp.*<br>*- E. coli*	*- Blastocystis sp.*	*- Cysts*	*- Blastocystis sp.*<br>*- flagellates*	NEG	NEG	POS<br>(25.22)	POS<br>(34.47)	NEG	NEG	-	-	-
DF253	V	NEG	NEG	NEG	NEG	NEG	NEG	NEG	NEG	NEG	NEG	-	-	-
DF254	V	NEG	NEG	NEG	NEG	NEG	NEG	POS<br>(24.47)	NEG	NEG	NEG	-	-	-
DF255	V	NEG	NEG	NEG	NEG	NEG	NEG	POS<br>(36.19)	NEG	POS<br>(42.88)	POS<br>(31.95)	-	-	-
DF256	V	*- Blastocystis sp.*	NEG	NEG	*- Blastocystis sp.*	NEG	NEG	POS<br>(30.13)	NEG	NEG	NEG	-	-	-
DF257	II	*- D. fragilis*<br>*- E. coli*	*- Blastocystis sp.*<br>*- E. coli*<br>*- E. histolytica/dispr*	NEG	*- Blastocystis sp.*<br>*- E. coli*	NEG	NEG	POS<br>(28.47)	NEG	NEG	NEG	POS	-	-
DF258	III	*- Blastocystis sp.*<br>*- E. coli*	*- Blastocystis sp.*<br>*- E. hartmanni*	*- E. coli*	*- Blastocystis sp.*<br>*- E. coli*	NEG	NEG	POS<br>(25.20)	NEG	NEG	NEG	-	-	-
DF259	III	NEG	NEG	NEG	NEG	NEG	NEG	NEG	NEG	NEG	NEG	-	-	-
DF260	III	NEG	*- Blastocystis sp.*<br>*- E. coli*	NEG	NEG	NEG	NEG	POS<br>(31.33)	NEG	NEG	NEG	-	-	-
DF261	III	*- Blastocystis sp.*	*- Blastocystis sp.*<br>*- E. coli*	NEG	NEG	NEG	NEG	POS<br>(22.53)	NEG	NEG	NEG	-	-	-
DF262	III	*- Blastocystis sp.*	*- Blastocystis sp.*<br>*- E. coli*	NEG	NEG	NEG	NEG	POS<br>(28.66)	NEG	NEG	NEG	-	-	-
DF263	III	*- Blastocystis sp.*<br>*- Amoebas*	*- Blastocystis sp.*<br>*- E. histolytica/dispar ?*	NEG	NEG	NEG	NEG	POS<br>(27.62)	NEG	NEG	NEG	POS	-	-
DF264	V	*- Blastocystis sp.*	NEG	NEG	NEG	NEG	NEG	POS<br>(29.53)	NEG	NEG	NEG	-	-	-
DF265	III	*- Blastocystis sp.*<br>*- E. coli*	*- Blastocystis sp.*<br>*- E. coli*	*- E. coli*	*- E. coli*	NEG	NEG	POS<br>(27.47)	NEG	NEG	NEG	-	-	-
DF266	III	NEG	NEG	NEG	NEG	NEG	NEG	POS<br>(23.44)	NEG	NEG	NEG	-	-	-
DF267	III	NEG	NEG	NEG	NEG	NEG	NEG	POS<br>(34.68)	NEG	NEG	NEG	-	-	-

DF268	III	NEG	NEG	NEG	NEG	NEG	NEG	POS (30.05)	NEG	NEG	NEG	-	-	-
DF269	III	NEG	- *Blastocystis sp.*<br>- *E. coli*	NEG	NEG	NEG	NEG	POS (28.17)	NEG	NEG	NEG	-	-	-
